# Probiotics, Prebiotics, and Synbiotics Improve Uremic, Inflammatory, and Gastrointestinal Symptoms in End-Stage Renal Disease With Dialysis: A Network Meta-Analysis of Randomized Controlled Trials

**DOI:** 10.3389/fnut.2022.850425

**Published:** 2022-04-04

**Authors:** Zixian Yu, Jin Zhao, Yunlong Qin, Yuwei Wang, Yumeng Zhang, Shiren Sun

**Affiliations:** Department of Nephrology, Xijing Hospital, Air Force Military Medical University, Xi’an, China

**Keywords:** probiotic, prebiotic, synbiotic, network meta-analysis, end-stage renal disease (ESRD)

## Abstract

**Background:**

Probiotics, prebiotics, and synbiotics are three different supplements to treat end stage renal disease (ESRD) patients by targeting gut bacteria. The comprehensive comparison of the effectiveness of different supplements are lacking.

**Objectives:**

The purpose of this network meta-analysis (NMA) is to assess and rank the efficacy of probiotics, prebiotics, and synbiotics on inflammatory factors, uremic toxins, and gastrointestinal symptoms (GI symptoms) in ESRD patients undergoing dialysis.

**Methods:**

Randomized clinical trials were searched from the PubMed, Embase, and Cochrane Register of Controlled Trials databases, from their inception until 4 September 2021. Random-effect model were used to obtain all estimated outcomes in network meta-analysis (NMA). Effect estimates were presented as mean differences (Mean ± SD) with 95% confidence interval (CI). The comprehensive effects of all treatments were ranked by the surface under the cumulative ranking (SUCRA) probabilities.

**Results:**

Twenty-five studies involved 1,106 participants were included. Prebiotics were superior in decreasing Interleukin-6 (IL-6; SMD –0.74, 95% CI [–1.32, –0.16]) and tumor-necrosis factor-α (TNF-α; SMD –0.59, 95% CI [–1.09, –0.08]), synbiotics were more effective in declining C-reactive protein (CRP; SMD –0.69, 95% CI [–1.14, –0.24]) and endotoxin (SMD –0.83, 95% CI [–1.38, –0.27]). Regarding uremic toxins, prebiotics ranked highest in reducing indoxyl sulfate (IS; SMD –0.43, 95% CI [–0.81, –0.05]), blood urea nitrogen (BUN; SMD –0.42, 95% CI [–0.78, –0.06]), and malondialdehyde (MDA; SMD –1.88, 95% CI [–3.02, –0.75]). Probiotics were rated as best in alleviating GI symptoms (SMD: –0.52, 95% CI [–0.93, –0.1]).

**Conclusion:**

Our research indicated prebiotics were more effective in declining IL-6, TNF-α, IS, MDA, and BUN, synbiotics lowering CRP and endotoxin significantly, and probiotics were beneficial for alleviating GI symptoms, which may contribute to better clinical decisions. This study was registered in PROSPERO (Number: CRD42021277056).

**Systematic Review Registration:**

[http://www.crd.york.ac.uk/PROSPERO], identifier [CRD42021277056].

## Introduction

End-stage renal disease (ESRD) is the ending of the deteriorating progression of chronic kidney disease (CKD), with high morbidity and mortality (1), estimating that more than 5.439 million ESRD patients worldwide by 2030 (2). Dialysis arose in 1960 and became an innovative technology that prolonged the lives of millions of people by serving as a bridge to kidney transplantation (3). However, the incomplete removal of inflammatory factors and some uremic toxins by dialysis makes it imperative to find new therapeutic strategies.

Amount studies in humans and animals have shown the reciprocal causative relationship between gut dysbiosis and CKD (4–6). Uremic toxins are the major factors in the development of uremia and systematic inflammation is a hallmark character of CKD, which influences the prognosis and life quality of ESRD patients (7, 8). Gut dysbiosis may aggravate the severity of CKD by producing nephrotoxic toxins as well as local or (and) systemic inflammatory responses (9). Dialysis, as the crucial treatment for ESRD patients, may also impair both the composition of gut microbiota and the integrity of the intestinal barrier (10). Therefore, re-establishment of a balanced gut microbiota in ESRD patients undergoing dialysis is hypothesized to improve both metabolic and immune disorders and to achieve a better prognosis. Gastrointestinal disease is the most common complication in ESRD patients, mainly due to impaired gut barrier and reduced residual renal function, aggravating the anxiety and sadness emotion (11). Probiotic, prebiotic, and synbiotic supplements are beneficial approaches for the establishment of a balanced gut microbiota by repairing the intestinal mucosal barrier and preventing harmful organism expansion and translocation, which were widely used as promising adjuvant treatments to relieve inflammatory response, uremic toxins, gastrointestinal symptoms, and improve the prognosis of dialysis patients (1, 12). However, their efficacy in reducing various indexes has not yet been determined. For example, a meta-analysis from March et al. (13) evaluated the effects of pro/pre/syn-biotics and found a significant reduction in indoxyl sulfate (IS), whereas another meta-analysis (14) reported these supplements have potential benefits to decrease p-Cresyl sulfate (PCS) instead IS in hemodialysis patients. To our knowledge, the available evidence may not provide a consistent conclusion among the three supplements.

Although probiotics, prebiotics and synbiotics are similar adjuvant treatments targeting gut bacteria, they have different mechanisms and functions that need to be distinguished. A randomized controlled trial (RCT) (15) investigated the efficacy of synbiotics on C-reactive protein (CRP) in hemodialysis patients, which showed CRP level was significantly reduced. In contrast, the outcomes from Shariaty et al. (16) suggested probiotic supplements had no obvious effects on the decline of serum CRP level in hemodialysis patients. Thus, distinguishing the efficacy of different supplements in dialysis patients is of great significance.

Taking into account all the above evidence, the efficacy of pro/pre/syn-biotics in ESRD patients among inflammatory factors, uremic toxins, and gastrointestinal (GI)- symptoms is still controversial. In addition, conditional pairwise meta-analysis failed to compare the associated merits among probiotics, prebiotics and synbiotics, which did not provide the best choice for the application of supplements targeting gut bacteria. Network meta-analysis (NMAs) not only improves the estimation accuracy of traditional meta-analysis but also indirectly compares interventions that were not compared in the original study. Thus, we conducted a comprehensive NMA to explore the efficacy of pro/pre/syn-biotic supplements in terms of inflammation, uremic toxins, and GI symptoms in dialysis patients, and ranked all interventions to find optimal supplements regarding different indexes, which definitely provided a prospective strategy in the future.

## Methods

This network meta-analysis was registered in the International Prospective Register of Systematic Reviews (Number: CRD42021277056), and completed based on the statements of the Preferred Reporting Items for Systematic Reviews and Meta Analyses (PRISMA).

### Literature Source and Searches

Two investigators (Z.X.Y and J.Z) independently performed an extensive search from PubMed, Embase and the Cochrane Register of Controlled Trials databases from their inception until September 4, 2021, Medical Subject Headings (MeSH) or free words of the keywords, including end-stage renal disease, dialysis, all spellings of known probiotic, prebiotic, synbiotic and randomized clinical trials (RCTs) were applied for document retrieval (the search strategy is shown in the Supplementary Table 1). Additional eligible studies from the identified studies and relevant system reviews, without the limitations of languages were screened. Inclusion criteria: (1) Randomized parallel or cross-over controlled trials on human subjects. (2) The participants were diagnosed with ESRD (≥18 years old) undergoing dialysis according to Kidney Disease: Improving Global Outcomes (KDIGO) 2021 Clinical Practice Guideline. (3) Patients in intervention groups received probiotic, prebiotic or synbiotic supplements for any dose or duration, and in any form (capsule, tablet, or other food supplements). (4) Patients in control groups received placebo or control therapy (defined as conventional therapy that specific drugs were not provided). (5) Studies reported at least one of the following outcomes: C-reactive protein (CRP), Interleukin-6 (IL-6), tumor necrosis factor-α (TNF-α), endotoxin, indoxyl sulfate (IS), p-Cresyl sulfate (PCS), indole-3-acetic acid (IAA), malondialdehyde (MDA), blood urea nitrogen (BUN), urea, creatinine, uric acid, and gastrointestinal (GI)-symptoms. Studies were excluded if: (1) Participants with functioning kidney transplant or CKD without dialysis. (2) Unavailable data of outcomes after contacting authors.

### Data Extraction and Quality Assessment

Two investigators (Z.X.Y and J.Z) independently extracted key data from the included studies using a standardized form to obtain study data in accordance with the Cochrane handbook. A third reviewer (S.R.S) resolved any conflicts in data extraction. The following information was extracted from each study: author, year of publication, baseline characteristics of participants (country, age, and sex), trial duration, and interventions. According to the Consensus statement of the International Association for Probiotic and Prebiotic Science (ISAPP) on probiotic and prebiotic (17, 18), prebiotics includes resistant starch, wheat flour, high-amylose corn starch, unripe banana flour, inulin-type fructans and probiotics includes *Lactobacillus acidophilus, Lactobacillus casei, Lactobacillus pentosus, Lactococcus lactis, Lactobacillus rhamnoses, Lactobacillus salivarius, Lactobacillus bulgaricus, Bifidobacterium bifidum, Bifidobacterium longum, Streptococcus thermophiles*, and *E. faecalis*. The Cochrane risk of bias tool was applied to assess the risk of all included studies based on different quality domains.

### Data Synthesis and Statistical Analyses

Stata (version14.0) and Revman statistical software (version 5.3) were employed to perform this NMA. In pairwise meta-analysis, fixed-effects model was used to pool the standard deviation of mean (SMD) and 95% confidence interval (CI) with low heterogeneity while random-effects model was used with moderate and high heterogeneity. According to the Cochrane handbook, heterogeneity was assessed using the Cochrane Q test and *I*^2^ statistics. *I*^2^ will be considered low heterogeneity (<40%), moderate heterogeneity (40–70%) and high heterogeneity (>70%) (19). A sensitivity analysis would be performed to ascertain the results of the NMA by excluding each of the individual studies. Funnel plot asymmetry and Egger’s regression test were used to verify Publication bias. The *P*-value < 0.05 suggested publication bias.

In indirect meta-analysis, the random-effects model and a Bayesian network meta-analysis approach were used to compare the effects of interventions. Inconsistency factors (IF) in closed loops were used to verify local consistency of the hypothesis. Effective estimates were presented as a standardized mean difference (SMD) with a 95% confidence interval (95% CI). These findings were interpreted as irrelevant when 95% of CI contained null values.

Network diagrams were used to show all treatment comparisons. The surface under the Cumulative Ranking Curve (SUCRA) was performed to measure the rank of the efficacy of all treatments. SUCRA is expressed as a percentage in reducing the changes of all outcomes, ranging from 0 to 1. A higher probability of SUCRA indicates a preference for the best treatment.

## Results

### Study Selection and Characteristics

**FIGURE 1:**
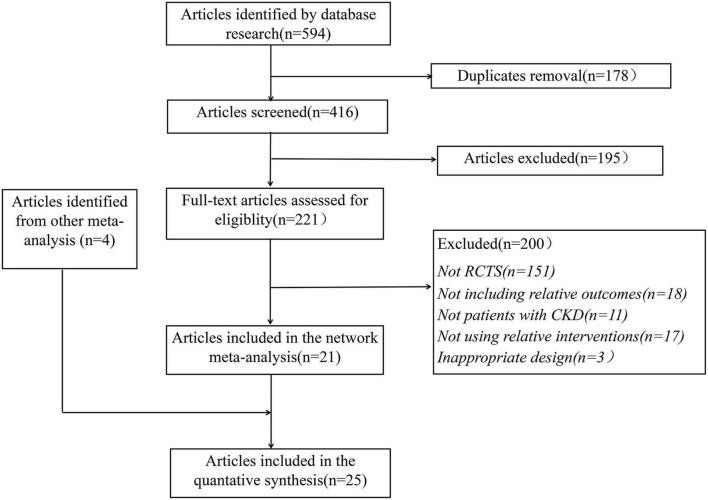
Flow Diagram for searching, identifying, screening, and qualifying for inclusion in randomized clinical trials.

Twenty-five RCT articles (15, 16, 20–42) with 1,106 participants were selected from 598 studies, the details of the study selection are shown in Figure 1.

These trials evaluated 4 different treatment agents, including probiotics, prebiotics, synbiotics, and placebo. Out of 25 studies, nine studies (22, 25, 26, 30, 32, 34, 36, 39, 40) compared prebiotics with placebo, nine studies (16, 21, 24, 27–29, 37, 41, 42) compared probiotics with placebo, six studies (15, 20, 23, 33, 35, 38) compared synbiotics with placebo as well as one study (31) was three-arm RCT that compared probiotics, synbiotics and placebo.

**FIGURE 2:**
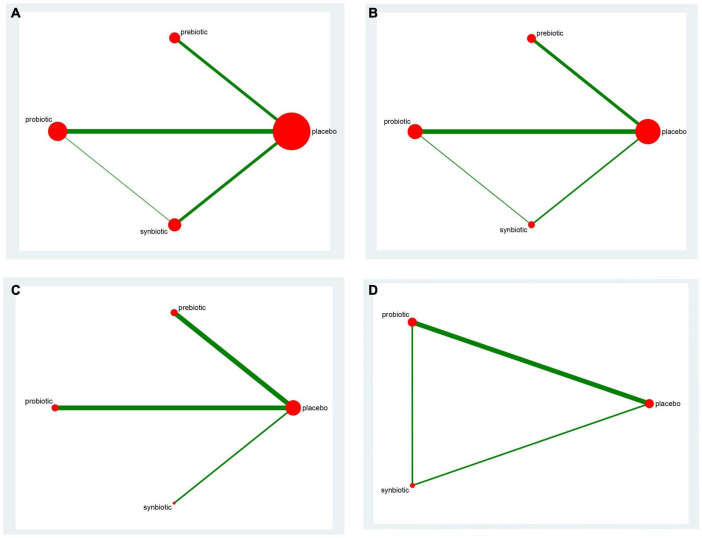
The network graph of all treatments of inflammatory factors. Outcome: **(A)** C-reactive protein (CRP); **(B)** Interleukin- 6 (IL-6); **(C)** tumor necrosis factor-α (TNF-α); **(D)** endotoxin. The number of studies for each treatment can be indicated by the size of each circle. Direct comparisons of tests can be expressed by lines between nodes, and the number of tests connected to the network can be expressed by the thickness of the lines.

The mean age of patients ranged from 28.84 to 71.20 years, and the time of intervention varied from 3 to 32 weeks. The baseline characteristics were shown in Table 1. The networks of eligible comparisons in available trials including inflammatory factors, uremic toxins, and GI symptoms were exhibited in Figure 2 and Supplementary Figures 7–9.

**TABLE 1:** Characteristics of included Interventions in dialysis patients.

		Sample (N)						Sex		
Study	Country	I	C	RCT design (blinding)	Patient	Intervention	During	M	F	Age (y, mean ± SD)
Esgalhado et al. (30)	Brazil	15	16	Randomized, double-blind, placebo- controlled trial	HD	I1: Prebiotic cookies (Resistant starch, Hi-Maize 260, Ingredion, United States), 16 g/d C1: Placebo cookies (manioc flour, Yoki), 16 g/d	4 w	18	13	I1:56.0 ± 7.5 C1:53.5 ± 11.5
Laffin et al. (34)	Canada	9	11	Randomized, double-blind, placebo-controlled parallel trial	HD	I1: Prebiotic biscuits (HAM-RS2 Ingredion ANZ Pty Ltd Lane Cove, NSW, Australia), 20 g/d C1: Regular wheat flour, 20 g/d	8 w	13	7	I1:53.8 ± 11.8 C1:57.6 ± 9
Meksawan et al. (26)	Thailand	9	9	Randomized, double-blind, placebo-controlled crossover trial	PD	I1:Prebiotic (fructo-oligosaccharides), 20 g/d C1:Sucrose, 20 g/d	4 w	5	4	I1:71.2 ± 6.5 C1:NA
Sirich et al. (22)	America	20	20	Randomized, single-blinded trial	HD	I1: Prebiotic corn (high-amylose corn starch, Hi-maize 260), 15 g/d C1: Waxy corn starch (AMIOCA), 15 g/d	6 w	24	16	I1:54 ± 14 C1:58 ± 13
Xie et al. (25)	China	39	44	Randomized controlled trial	HD	I1: Prebiotic fiber, 20 g/d C1: Placebo starch, 20 g/d	6 w	44	38	I1:51.7 ± 15.7 C1:53.1 ± 13.2
De Andrade et al. (40)	Brazil	26	26	Randomized, double-blind, placebo- controlled crossover trial	PD	I1: Prebiotic flour (Unripe Banana Flour), 21 g/d C1: Placebo sachets (6 g waxy corn starch), 21 g/d	12 w	14	12	I1:55 ± 12 C1:NA
Biruete et al. (39)	Iran	12	12	Randomized, double-blind, placebo-controlled, crossover trial	HD	I1: Prebiotic (inulin: females: 10 g/day; males: 15 g/day) C1: Maltodextrin (females: 6 g/day; males: 9 g/day)	12 w	6	6	I1:55 ± 10 C1:NA
Li et al. (36)	China	15	15	Randomized, double-blind, placebo- controlled, crossover trial	PD	I1: Prebiotic (inulin-type fructans), 10 g/d C1: Placebo, 10 g/d	12 w	6	9	I1:28.84 ± 38.14 C1:NA
Khosroshahi et al. (32)	Iran	23	21	Randomized double-blind controlled clinical trial	HD	I1: Prebiotic crackers(20 g or 25 g of 60% resistant starch) C1: Placebo crackers (20 g or 25 g of waxy corn starch)	32 w	29	21	I1:53.17 ± 10.15 C1:57.9 ± 13.34
Lim et al. (41)	China	25	25	Randomized double- blind placebo-controlled clinical trial	HD	I1: Probiotic sachets (Lactococcus lactis subsp. Lactis LL358, Lactobacillus salivarius LS159, and Lactobacillus pentosus LPE588 at high dose, 100 billion; 13 × 10^11^ cfu/day), 6 g/d C1: Placebo sachets, 6 g/d	24 w	20	30	I1: 61.50 ± 10.30 C1:56.28 ± 12.36
Soleimani et al. (27)	Iran	30	30	Randomized double-blind placebo-controlled parallel clinical trial	HD	I1: Probiotic capsule (L. acidophilus, L casei and B. bifidum)2 10^9^ CFU/g /d CI: Placebo	12 w	40	20	I1: 54 ± 16 C1: 59.4 ± 16
Wang et al. (24)	China	21	18	Randomized, double-blind, placebo-controlled trial	PD	I1:Probiotic capsule, 90 billion CFU/day C1: Placebo capsule (maltodextrin)	24 w	18	21	I1: 51 ± 11.33 C1: 53.5 ± 11.85
Borges et al. (28)	Brazil	16	17	Randomized, double-blind, placebo-controlled trial	HD	I1: Probiotic capsule (30 billion live bacteria, totalizing 90 billion colony-forming units (CFU)/d, included Streptococcus thermophilus, Lactobacillus acidophilus, and Bifidobacterial longum), 3 capsules/d C1: Placebo capsule, 3 capsules/d	12 w	21	12	I1: 53.6 ± 11.0 C1: 50.3 ± 8.5
Liu et al. (37)	China	22	23	Randomized double-blind placebo trial	HD	I1: Probiotic capsule (2.2 × 10^9^ cfu Balonium NQ1501, 0.53 × 10^9^ cfu.L. acidophilus YIT2004, and 1.1 × 10^9^ cfu E. faecalis YIT0072), 8 capsule/d C1: Placebo capsules (pregelatinized starch and lactose), 8 capsule/d	24 w	23	22	I1:49 ± 9 C1:48 ± 11
Pan et al. (42)	China	50	48	Randomized controlled trial	PD	I1: Probiotic capsules (Bifidobacterium longum, Lactobacillus bulgaricus, and Streptococcus thermophilus), 6 capsules/d C1: Maltodextrin capsules, 6 capsules /d	8 w	56	42	I1: 49.31 ± 13.13 C1:50.92 ± 17.60
Natarajan et al. (21)	America	19	18	Randomized, double-blind, placebo-controlled crossover trial	HD	I1: Probiotic capsule (30 billion CFU of S. thermophilus KB 19, L. acidophilus KB 27, and B. longum KB 31), 6 capsules/d C1: Placebo capsules (a 1:1 blend of cream of wheat and psyllium husk)/d	24 w	6	16	I1:54 ± 39.62 C1:NA
Eidi et al. (29)	Iran	21	21	Randomized triple -blind placebo-controlled trial	HD	I1:Probiotic capsule (1.6 × 10^7^ CFU of Lactobacillus Rhamnoses), one capsule/d C1: Placebo capsule, 1 capsule/d	4 w	32	10	I1: 57.05 ± 13.95 C159.67 ± 15.04
Soleimani et al. (15)	Iran	30	30	Randomized, double-blind, placebo-controlled clinical trial	HD	I1: Synbiotic capsule (Lactobacillus acidophilus, Lactobacillus casei, and Bifidobacterium bifidum (2 × 10^9^ CFU/day each) plus 0.8 g/day of inulin) CI: Placebo (corn starch)	12 w	42	18	I1: 62.8 ± 12.7 C1: 62.8 ± 14.8
Viramontes-Horner et al. (23)	Mexico	20	15	Randomized double-blind, placebo-controlled, clinical trial	HD	I1: Symbiotic gel (Nutrihealth, Nutriments Inteligents, S.A. de C.V, Guadalajara, Jalisco, Mexico) contained a mix of probiotics and 2.31 g of a prebiotic fiber (inulin); 1.5 g of omega-3 fatty acids and vitamins), 14 gels/d CI: Placebo, 14 gels/d	8 w	32	10	I1: 40.6 ± 17.1 C1: 39.0 ± 16.0
Lopes et al. (35)	Brazil	29	29	Randomized, simple-blind, placebo-controlled trial	HD	I1: Synbiotic drink (100 ml probiotic and 40 g of extruded sorghum flakes) C1: Placebo drink (100 mL of pasteurized milk and 40 g of extruded corn flakes)	7 w	38	20	I1:63.17 ± 11.16 C1:63.03 ± 10.77
Haghighat et al. (31)	Iran	I1:23 I2:23	19	Randomized, double-blind, parallel group, placebo-controlled trial	HD	I1: Synbiotic sachet (5 g probiotics and 15 g of prebiotics), 20 g/d I2: Probiotic powder (5 g probiotics and 15 g of maltodextrin powder), 20 g/d C1: Maltodextrin powder, 20 g/d	12 w	34	31	I1: 48.04 ± 10.11 I2: 46.21 ± 11.49 C1:45.47 ± 10.76
Kooshki et al. (33)	Iran	23	23	Randomized, double-blind, placebo-controlled trial	HD	I1: Synbiotic capsules (100 mg of lactol probiotic, which contains Lactobacillus coagulant and fructo-oligosaccharides), 2 capsules/d C1: Placebo capsules (farina), 2 capsules/d	8 w	21	25	I1: 62.92 ± 16.80 C1:62.83 ± 16.62
Cruz-Mora et al. (20)	Mexico	8	10	Randomized, double-blind, placebo-controlled clinical trial	HD	I1: Symbiotic gel (probiotic of 2.0 3 × 10^12^ colony-forming units; 2.31 g of a prebiotic fiber (inulin); 1.5 g of omega-3 fatty acids (eicosatetraenoic and docosahexaenoic acid) and vitamins (complex B, folic acid, ascorbic acid, and vitamin E) C1: Placebo gel (a gel without prebiotic fiber, probiotics, omega-3 fatty acids, and vitamins)	8 w	15	3	I1:34 ± 10 C1:30.6 ± 9.5
Mirzaeian et al. (38)	Iran	21	21	Randomized, double-blind, placebo-controlled clinical trial	HD	I1: Synbiotic capsule (Lactobacillus casei L. acidophilus Rhamnoses, Bulgaricus, Bifidobacterium breve, B. longum and Streptococcus thermophiles and fructo-oligosaccharide as prebiotic in addition to lactose, magnesium stearate, and talc as filling materials), 1 g/d CI: Placebo capsules (maltodextrin), 1 g/d	8 w	30	12	I1:58.30 ± 11.3 C1:69.74 ± 42.87

*I, intervention; C, control; RCT, randomized clinical trial; HD, hemodialysis; PD: peritoneal dialysis; M, male; F, female; W, week; NA, not available.*

### Risk of Bias and Quality Assessment

Among the 25 studies, 15 studies (15, 16, 22, 24, 27, 29, 31, 32, 36–42) mentioned the method of random sequence generation and were rated as “low risk,” 11 articles (15, 16, 24, 27, 28, 31, 37, 39–42) referred to allocation concealment, were rated as “low risk,” 16 articles (15, 16, 24, 27–32, 34, 36–41) reported how the Blinding of participants and personnel performed, and 14 studies (15, 16, 23, 24, 27, 28, 30–32, 36–38, 40, 41) reported the Blinding of outcome assessment. Supplementary Figures 1, 2 summarized the all risks of bias.

### Network Meta-Analysis

#### Inflammatory Factors

**FIGURE 3:**
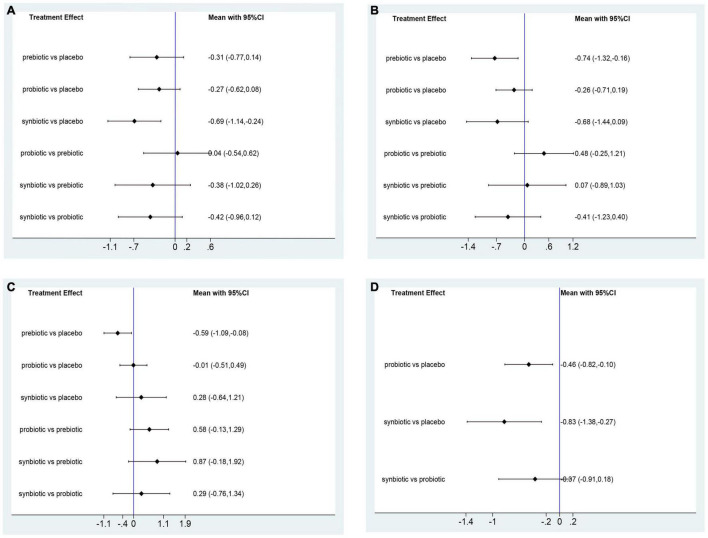
Forest plots of network meta-analysis of inflammatory factors. Forest plots of the network meta-analysis of the effect of all supplementations on **(A)** C-reactive protein (CRP, mg/dl); **(B)** Interleukin- 6 (IL-6, pg/ml); **(C)** tumor necrosis factor-α (TNF-α, pg/ml); **(D)** endotoxin (IU/L).

**FIGURE 4:**
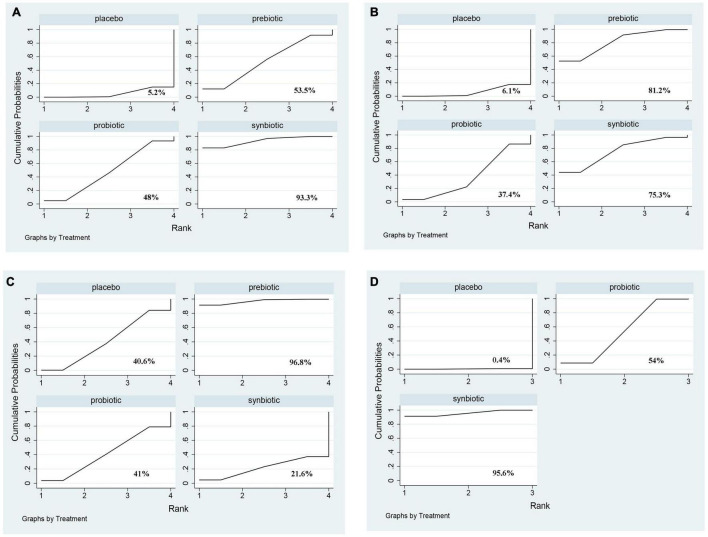
The cumulative ranking area of inflammatory factors. Treatment strategies were ranked based on their probability of reducing **(A)** C-reactive protein (CRP); **(B)** Interleukin- 6 (IL-6); **(C)** tumor necrosis factor-α (TNF-α); **(D)** endotoxin by cumulative ranking area (SUCRA). The greater the probability, the better the effect.

In this network meta-analysis, synbiotics lowered CRP level (SMD –0.69; 95% CI [–1.14, –0.24]), prebiotics decreased IL-6 level (SMD –0.74; 95% CI [–1.32, –0.16]) and TNF-α (SMD –0.59; 95% CI [–1.09, –0.08]). Probiotics and synbiotics declined the concentration of endotoxin (probiotics: SMD –0.46; 95% CI [–0.82, –0.10], synbiotics: SMD –0.83; 95% CI [–1.38, –0.27]) (Figure 3 and Supplementary Figure 10). Synbiotics ranked as the best intervention in the reduction of CRP (SUCRA = 93.3%) and endotoxin (SUCRA = 95.6%). Prebiotics were rated as the first supplements in declining IL-6 (SUCRA = 81.2%) and TNF-α (SUCRA = 96.8%) (Figure 4).

#### Uremic Toxins

**FIGURE 5:**
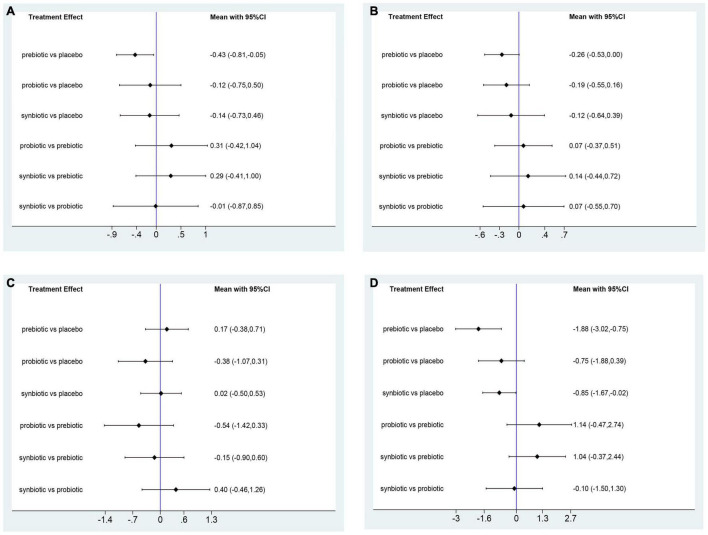
Forest plots of network meta-analysis of uremic toxins. Forest plots of the network meta-analysis of the effect of all supplementations on **(A)** indoxyl sulfate (IS, mg/L); **(B)** p-cresyl sulfate (PCS, mg/L); **(C)** indole-3-acetic acid (IAA, μmol/L); **(D)** malondialdehyde (MDA, μmol/L).

**FIGURE 6:**
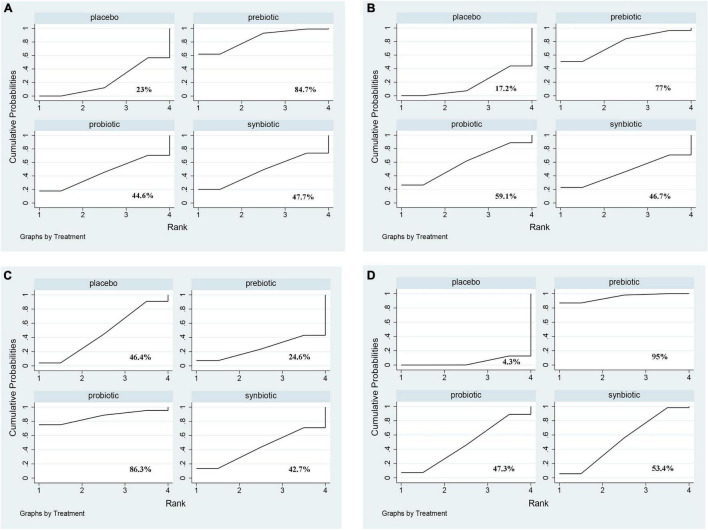
The cumulative ranking area of uremic toxins; Treatment strategies were ranked based on their probability of reducing **(A)** indoxyl sulfate (IS); **(B)** p-cresyl sulfate (PCS); **(C)** indole-3-acetic acid (IAA); **(D)** malondialdehyde (MDA) by cumulative ranking area (SUCRA). The greater the probability, the better the effect.

Uremic toxins including IS, PCS, IAA, and MDA were evaluated. The outcome revealed prebiotics were superior in declining IS (prebiotics: SMD −0.43; 95% CI [−0.81, −0.05]), prebiotics and synbiotics were effective supplements on the alteration of MDA level (prebiotics: SMD −1.88; 95% CI [−3.02, −0.75]; synbiotics: SMD −0.85; 95% CI [−1.67, −0.02]) but no supplements significantly declined serum PCS, and IAA (Figure 5 and Supplementary Figure 11). With regard to IS, PCS, and MDA, prebiotics were ranked as the first therapeutic option, where the SUCRA were 84.7, 77, and 95%, respectively. Probiotics had the highest possibility in serum IAA level (SUCRA = 86.3%) (Figure 6).

#### GI Symptoms

**FIGURE 7:**
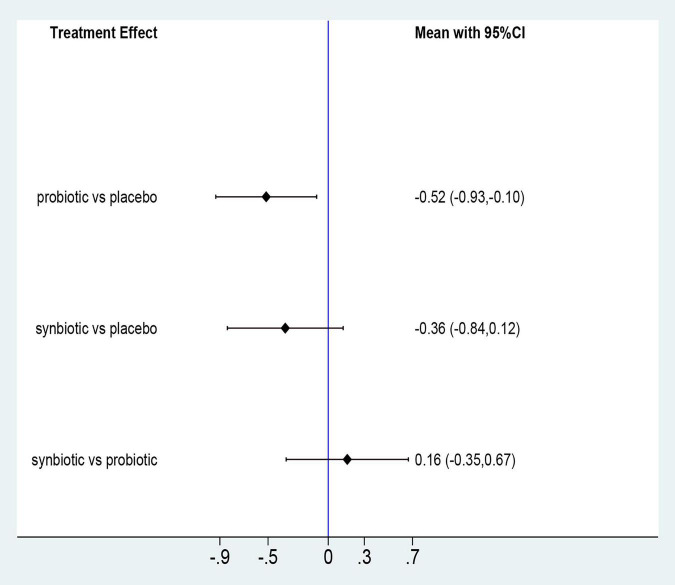
Forest plots of network meta-analysis of GI symptoms; Forest plots of the network meta-analysis of the effect of all supplementations on gastrointestinal-symptoms (GI symptoms).

**FIGURE 8:**
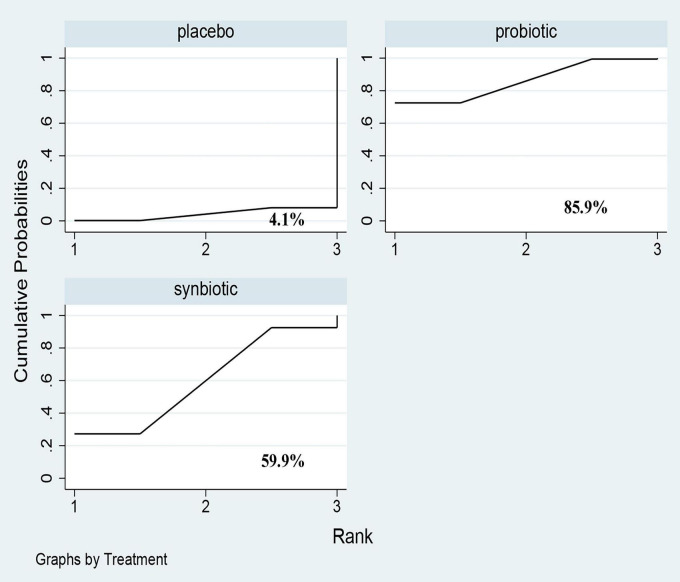
The cumulative ranking area of GI symptoms; Treatment strategies were ranked based on their probability of reducing gastrointestinal-symptoms (GI symptoms) by cumulative ranking area (SUCRA). The greater the probability, the better the effect.

Three original RCTs utilize Gastrointestinal Symptom Rating Scale (GSRS) to assess GI symptoms. Probiotic supplement (SMD –0.52; 95% CI [–0.93, –0.1]) exhibited significant remission in GI symptoms (Figure 7 and Supplementary Figure 12). Probiotics were rated as the best treatment in alleviating GI symptoms (SUCRA = 85.9%), the second rank was synbiotics (SUCRA = 59.9%), and the last was placebo (SUCRA = 4.1%) (Figure 8).

#### Other Clinical Outcomes

**FIGURE 9:**
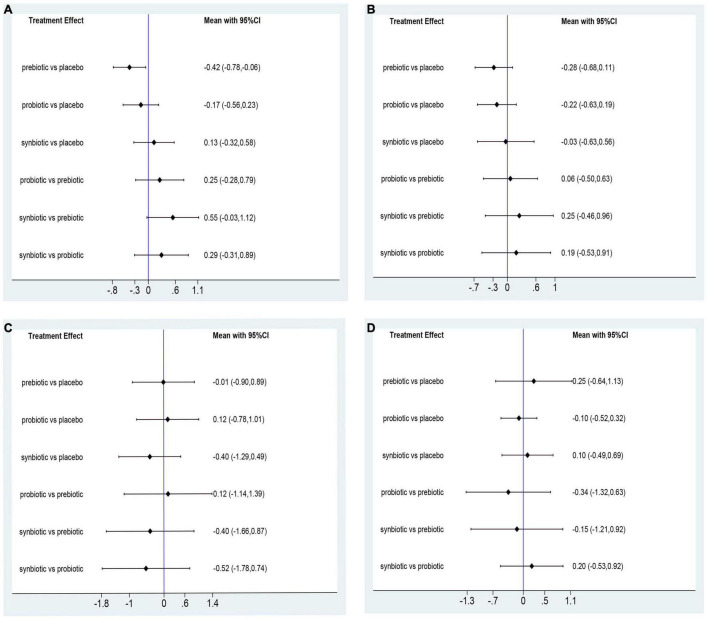
Forest plots of network meta-analysis of other clinical outcomes. Forest plots of the network meta-analysis of the effect of all supplementations on **(A)** BUN (mg/dl); **(B)** creatinine (mg/dl); **(C)** urea (mg/dl); **(D)** uric acid (mg/dl).

**FIGURE 10:**
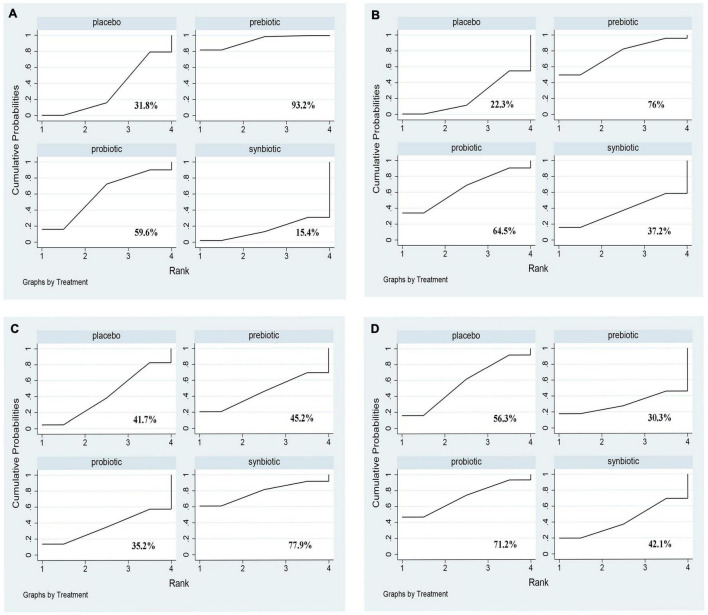
The cumulative ranking area of other clinical outcomes; Treatment strategies were ranked based on their probability of reducing **(A)** BUN (mg/dl); **(B)** creatinine (mg/dl); **(C)** urea (mg/dl); **(D)** uric acid (mg/dl) by cumulative ranking area (SUCRA). The greater the probability, the better the effect.

Comparative analyses of other clinical outcomes including BUN, creatinine, urea and uric acid were shown in Figure 9 and Supplementary Figure 13. A significant reduction of BUN was found after providing prebiotics (SMD –0.42; 95% CI [–0.78, –0.06]). No supplements were significantly in decreasing serum creatinine, urea and uric acid. Prebiotics lowering BUN (SUCRA = 93.2%) and creatinine (SUCRA = 76%) effectively, synbiotics had the highest possibility in the change of urea (SUCRA = 77.9%), whereas probiotics were the superior treatment in declining uric acid level (SUCRA = 71.2%) (Figure 10).

### Primary Analysis

Compared to placebo, the three drugs declined CRP, IL-6, IS, PCS, MDA, creatinine, endotoxin, and GI symptoms significantly (Supplementary Figures 3–6). Publication bias were examined by funnel plot and Egger’s test, and no publication bias were found in any indexes (Supplementary Figure 14 and Supplementary Table 2).

### Inconsistency Analysis

There is one closed loop in this network meta-analysis comparing the supplementation of probiotics, synbiotics, and placebo in the changes of CRP, IL-6, endotoxin, and GI symptoms. *P*-values > 0.05 indicates that direct and indirect estimates are consistent. There is no significant difference between direct estimation and indirect estimation (Supplementary Table 3).

### Sensitivity Analysis

Sensitivity analysis were performed when moderate and high homogeneity of primary outcomes were found. After removing studies one by one, the outcomes were similar to the original studies among CRP, IL-6, IS, MDA, and urea. But the three supplements were no significantly difference in reducing TNF-α after excluding the original study of Laffin et al. (34) and Xie et al. (25) (Supplementary Figure 15).

## Discussion

This is the first NMA to evaluate the efficacy of pro/pre/syn-biotic supplements among ESRD patients undergoing dialysis and rank the efficacy of the three treatments to find the best choice, which is more important in clinical practice, but has not been determined. In the pairwise meta-analysis, the three supplements significantly declined CRP, IL-6, IS, PCS, MDA, creatinine, endotoxin, and GI symptoms compared to placebo. In this NMA, regarding inflammatory factors, prebiotics were rated as best in reducing IL-6 and TNF-α, and synbiotics were superior in reducing CRP and endotoxin; in terms of uremic toxins, prebiotic supplements were more effective in decreasing IS, MDA, and BUN; probiotics ranked highest in attenuating GI symptoms.

Concerning inflammatory factors, prebiotics ranked highest in declining IL-6 and TNF-α level, and synbiotics were superior in diminishing CRP and endotoxin. Systemic persistent inflammation is associated with reduced renal function, fluid imbalance, and immune dysfunction in ESRD, and dialysis, as the main treatment for ESRD patients, also exacerbated the occurrence of persistent inflammation (43). Although the etiology of inflammation was regarded as diversified in ESRD patients, the role of intestinal microbiota has been recognized under the theory of the gut-kidney axis (44). Wang et al. (45) performed evidence that intestinal bacterial translocation led to the increase of serum IL-6 and CRP levels in uremic rats, facilitating the progression of ESRD. As a non-invasive adjuvant intervention, pro/pre/syn-biotics have been demonstrated to attenuate inflammation response in previous meta-analysis (13), coinciding with our results. In addition, we further confirmed that the optimal supplements might be used to treat different inflammatory cytokines specifically. Vaziri et al. (46) found the activity of the nuclear factor Kappa-B (NF-κB) which mediated the transcription of IL-6 gene was decreased in nephrectomized rats after the administration of prebiotics, indicating the crucial role of prebiotics in attenuating IL-6. Synbiotics significantly declined Endotoxin by alleviating the inflammatory response triggered by CD14 and Toll-like receptor interactions (47, 48). The above research has shown strong evidence supporting the beneficial effects of different supplements in ESRD patients.

Prebiotics were superior in reducing serum IS, prebiotics were rated as best in reducing MDA level. The accumulation of metabolic toxins in the blood is closely associated with the deteriorating progression of CKD to ESRD, part of the toxins, such as protein-bound uremic toxins, come from intestinal flora, and dialysis is not potentially removed (9, 45). The efficacy of pro/pre/syn-biotics in lowering uremic toxins has been demonstrated by previous meta-analysis (13, 49). Our pairwise comparison found the same results and notably we further suggested that prebiotics and synbiotics are the most effective supplements. Prebiotics are some non-digestible food ingredients, regarded as a vital dietary supplement for ESRD patients with dietary restriction of protein intake, increasing the concentration of short-chain fatty acids (SCFAs), which benefit metabolites produced by gut bacterium (12, 50). Decreased SCFAs were regarded as one of the main mechanisms of the production of uremic toxins, which may also be the reason why prebiotics were more effective than probiotics and synbiotics. MDA is a low-molecular-weight solution that participates in oxidative stress, connecting with the progress of CKD and its cardiovascular complications (51). Seven randomized controlled trials were introduced in the study of Nguyen et al. (14), who found that MDA was significantly reduced in hemodialysis patients after taking three supplements. Several studies also have demonstrated that synbiotics might increase the expression of the antioxidant gene SOD and GPX in the gut by targeting gut bacteria to activate oxidative stability (52, 53). Current studies support the evidence that taking prebiotics and synbiotics have the most beneficial influence in reducing IS and MDA. Whereas, it is of great importance to emphasize that the change of uremic toxins is the result of multiple comparisons among the three drugs, combining small samples of studies and different follow-up times, which declined the strength of evidence, contributing to the accuracy of evidence is low. Thus, launching large clinical trials is important to evaluate the function of pro/pre/syn-biotics in reducing uremic toxins, especially protein-bound uremic toxins.

We investigated other clinical parameters including BUN, creatinine, urea, and uric acid that are related to kidney function, showing prebiotics ranked highest in declining serum BUN level. Nevertheless, no supplements obviously decreased creatinine, urea, and uric acid level. The diffusion of circulating BUN into the intestinal lumen is conducive to the growth of intestinal bacteria expressing urease and producing uremia toxin, leading to the destruction of the intestinal barrier, thereby promoting systemic inflammation (54). Prebiotics may accelerate the excretion of BUN from bloodstream by decreasing protein degradation and indirectly removing inflammatory factors (55). Under normal circumstances, a small part of creatinine, urea, and uric acid can also be excreted through the intestine to maintain homeostasis. However, the gut dysbiosis of ESRD patients interferes with the excretion of these toxins, resulting in the accumulation of uremic toxins and further deterioration of renal function (47). In line with our NMA result, Laffin et al. (34) found that resistant starch (a prebiotic) could increase the bacterial families possessing urease, uricase in ESRD patients when compared with health control. On the contrary, a system review launched by March et al. (13) found these supplements had no significant difference in reducing BUN compared to health control, the inconsistent outcome may be due to that study regarding probiotics, prebiotics, and synbiotics as one supplement which reduced the accuracy of the results.

Approximately 32–85% of patients suffered from GI disorders, tending to present a higher prevalence of diarrhea, abdominal pain, constipation and so on, causing both physical and psychological burden on ESRD patients (11, 37). Three original RCTs utilized Gastrointestinal Symptom Rating Scale (GSRS) (20, 37, 56) to assess GI symptoms, which found probiotics were the best supplements. Numerous diseases including Coronavirus Disease (COVID-19), Irritable Bowel Syndrome (IBD), and kidney disease, which are associated with gastrointestinal disorders, have found that the disruption of gut bacteria may play an important role (57, 58). Probiotic strains significantly activate the aromatic hydrocarbon receptor (AhR) way to stimulate the intestinal immune system to relieve GI symptoms (59). Dimidi et al. (60) revealed that administration of probiotics decreased gut transit time, increased stool frequency, and improved some constipation-related symptoms. Although our studies discovered that probiotics can significantly improve gastrointestinal symptoms, the number of RCTs involved is limited leading to the high homogeneity of results which allowed us to perform further research.

This is the first network meta-analysis to explore the efficacy of probiotics, prebiotics, and synbiotics in ESRD patients, obtaining the rank of all supplements in terms of inflammatory factors, uremic toxins, and GI symptoms, which may provide a prospective viewpoint for clinical practice. Moreover, limiting study designs to RCT helps us to obtain robust outcomes. But some limitations should pay attention to: First, since few multi-arms were included in this meta-analysis, most of the original studies were two-arm studies with placebo, and the number of original RCTs used to evaluate MDA, endotoxin, and GI symptoms were small, leading to slightly poor accuracy of the results. Second, probiotics, prebiotics, and synbiotics are composed of a variety of beneficial bacteria or substances, but we have not been able to compare the specific bacterium or components that contribute to the efficacy, internal confounding factors and heterogeneity cannot be avoided. Third, it cannot be ruled out that some variations in route of administration, dose, duration of intervention in each study may result in different outcomes. Therefore, high-quality international studies are still needed to confirm this conclusion.

## Conclusion

This NMA demonstrated that prebiotics were superior in declining IL-6, TNF-α, IS, MDA, and BUN, synbiotics ranked best in the decrease of CRP and endotoxin, and probiotics were the most effective supplements for alleviating GI symptoms in ESRD patients undergoing dialysis. Our study is the first to distinguish the three supplements and obtain an optimal treatment regimen for inflammation, uremic toxins, and gastrointestinal symptoms, which will provide a prospective strategy for the application of pro/pre/syn-biotics in ESRD patients in clinical practice.

## Data Availability Statement

The original contributions presented in the study are included in the article/Supplementary Material, further inquiries can be directed to the corresponding author.

## Author Contributions

ZY, JZ, and SS: conceptualization, writing—original draft, and writing—review and editing. ZY and JZ: methodology. ZY, JZ, YQ, and YW: software. YW and YZ: validation and visualization. ZY and YQ: supervision. All authors contributed an important role in drafting manuscript, accepting accountability, and ensuring the accuracy or completeness of the overall work are properly investigated and resolved.

## Conflict of Interest

The authors declare that the research was conducted in the absence of any commercial or financial relationships that could be construed as a potential conflict of interest.

## Publisher’s Note

All claims expressed in this article are solely those of the authors and do not necessarily represent those of their affiliated organizations, or those of the publisher, the editors and the reviewers. Any product that may be evaluated in this article, or claim that may be made by its manufacturer, is not guaranteed or endorsed by the publisher.

## Abbreviations

ESRD: End-stage renal disease
CRP: C-reactive protein
IL-6: Interleukin-6
MDA: malondialdehyde
TNF- α: tumor necrosis factor- α
IS: Indoxyl sulfate
PCS: p-Cresyl sulfate
IAA: Indole-3-acetic acid
BUN: blood urea nitrogen
GI symptoms: gastrointestinal symptoms
SUCRA: the surface under the cumulative ranking
CKD: chronic renal disease
RCTs: randomized controlled trials
KDIGO: Kidney Disease: Improving Global Outcomes
ISAPP: the International Association for Probiotic and Prebiotic Science
GSRS: Gastrointestinal Symptom Rating Scale.
