# Corrigendum: Probiotics, prebiotics, and synbiotics improve uremic, inflammatory, and gastrointestinal symptoms in end-stage renal disease with dialysis: A network meta-analysis of randomized controlled trials

**DOI:** 10.3389/fnut.2022.984187

**Published:** 2022-07-27

**Authors:** Zixian Yu, Jin Zhao, Yunlong Qin, Yuwei Wang, Yumeng Zhang, Shiren Sun

**Affiliations:** Department of Nephrology, Xijing Hospital, Air Force Military Medical University, Xi'an, China

**Keywords:** probiotic, prebiotic, synbiotic, network meta-analysis, end-stage renal disease (ESRD)

In the published article, there was an error in Figure 6 as published. The panel C (indole-3-acetic acid, IAA) and panel D (malondialdehyde, MDA) in Figure 6 is the same as the panel C (tumor necrosis factor-α, TNF-α) and panel D (endotoxin) in Figure 4. The corrected Figure 6 and its caption appear below.

**Figure 6:**
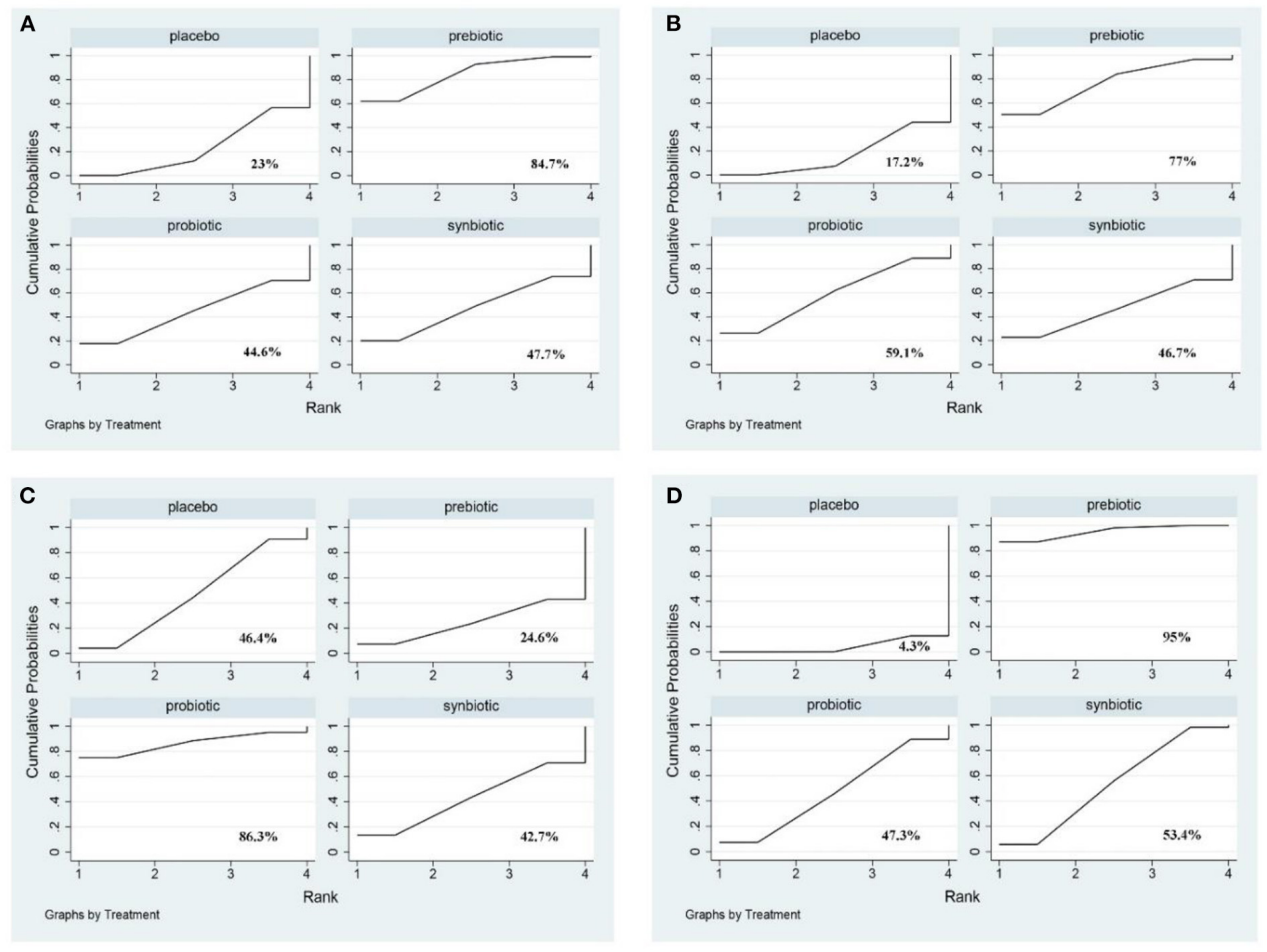
The cumulative ranking area of uremic toxins; Treatment strategies were ranked based on their probability of reducing **(A)** indoxyl sulfate (IS); **(B)** p-cresyl sulfate (PCS); **(C)** indole-3-acetic acid (IAA); **(D)** malondialdehyde (MDA) by cumulative ranking area (SUCRA). The greater the probability, the better the effect.

In the published article, there was an error in Table 1 as published. The number of males in Liu et al. is wrong, 28 should be changed to 23. The corrected Table 1 and its caption appear below.

**Table 1:** Characteristics of included Interventions in dialysis patients.

		**Sample**						**Sex**		
**Study**	**Country**	**I**	**C**	**RCT design (blinding)**	**Patient**	**Intervention**	**During**	**M**	**F**	**Age**
Esgalhado et al. (30)	Brazil	15	16	Randomized, double-blind, placebo-controlled trial	HD	I1: Prebiotic cookies (Resistant starch, Hi-Maize^®^ 260, Ingredion, USA), 16 g/d C1: Placebo cookies (manioc flour, Yoki), 16 g/d	4 w	18	13	I1:56.0 ± 7.5 C1:53.5 ± 11.5
Laffin et al. (34)	Canada	9	11	Randomized, double-blind, placebo-controlled parallel trial	HD	I1: Prebiotic biscuits (HAM-RS2 Ingredion ANZ Pty Ltd Lane Cove, NSW, Australia), 20 g/d C1: Regular wheat flour, 20 g/d	8 w	13	7	I1:53.8 ± 11.8 C1:57.6 ± 9
Meksawan et al. (26)	Thailand	9	9	Randomized, double-blind, placebo-controlled crossover trail	PD	I1: Prebiotic (fructo-oligosaccharides), 20 g/d C1: Sucrose, 20 g/d	4 w	5	4	I1:71.2 ± 6.5 C1:NA
Sirich et al. (22)	America	20	20	Randomized, single-blinded trial	HD	I1: Prebiotic corn (high-amylose corn starch, Hi-maize 260), 15 g/d C1: Waxy corn starch (AMIOCA), 15 g/d	6 w	24	16	I1:54 ± 14 C1:58 ± 13
Xie et al. (25)	China	39	44	Randomized controlled trial	HD	I1: Prebiotic fiber, 20 g/d C1:Placebo starch, 20 g/d	6 w	44	38	I1:51.7 ± 15.7 C1:53.1 ± 13.2
De Andrade et al. (40)	Brazil	26	26	Randomized, double-blind, placebo-controlled crossover trial	PD	I1: Prebiotic flour (Unripe Banana Flour), 21 g/d C1: Placebo sachets (6 g waxy corn starch), 21 g/d	12 w	14	12	I1:55 ± 12 C1:NA
Biruete et al. (39)	Iran	12	12	Randomized, double-blind, placebo-controlled, crossover trial	HD	I1: Prebiotic (inulin: females: 10 g/day; males: 15 g/day) C1: Maltodextrin (females: 6 g/day; males: 9 g/day)	12 w	6	6	I1:55 ± 10 C1:NA
Li et al. (36)	China	15	15	Randomized, double-blind, placebo-controlled, crossover trial	PD	I1: Prebiotic (inulin-type fructans), 10 g/d C1: Placebo, 10 g/d	12 w	6	9	I1:28.84 ± 38.14 C1:NA
Khosroshahi et al. (32)	Iran	23	21	Randomized double-blind controlled clinical trial	HD	I1: Prebiotic crackers (20 g or 25 g of 60% resistant starch) C1: Placebo crackers (20 g or 25 g of waxy corn starch)	32 w	29	21	I1:53.17 ± 10.15 C1:57.9 ± 13.34
Lim et al. (41)	China	25	25	Randomized double- blind placebo-controlled clinical trial	HD	I1: Probiotic sachets (Lactococcus lactis subsp. Lactis LL358, Lactobacillus salivarius LS159, and Lactobacillus pentosus LPE588 at high dose,100 billion; 13 ×10^11^cfu/day), 6 g/d C1: Placebo sachets, 6 g/d	24 w	20	30	I1: 61.50 ± 10.30 C1:56.28 ± 12.36
Shariaty et al. (16)	Iran	18	18	Randomized, double-blind, parallel group, placebo-controlled trial	HD	I1: Probiotic capsule (Lactobacillus acidophilus, Bifidobacterium and Streptococcus thermophilus (beneficial bacteria), 500 mg/d CI: Placebo, 500 mg/d	12 w	20	16	I1:54.17 ± 13.60 C1: 61.50 ± 8.68
Soleimani et al. (27)	Iran	30	30	Randomized double-blind placebo-controlled parallel clinical trial	HD	I1: Probiotic capsule (L. acidophilus, L casei and B. bifidum)2 10^9^ CFU/g/d CI: Placebo	12 w	40	20	I1: 54 ± 16 C1: 59.4 ± 16
Wang et al. (24)	China	21	18	Randomized, double-blind, placebo-controlled trial	PD	I1: Probiotic capsule, 90 billion CFU/day C1: Placebo capsule(maltodextrin)	24 w	18	21	I1: 51 ± 11.33 C1: 53.5 ± 11.85
Borges et al. (28)	Brazil	16	17	Randomized, double-blind, placebo-controlled trial	HD	I1: Probiotic capsule (30 billion live bacteria, totalizing 90 billion colony-forming units (CFU)/d, included Streptococcus thermophilus, Lactobacillus acidophilus, and Bifidobacterial longum), 3 capsules/d C1: Placebo capsule, 3 capsules/d	12 w	21	12	I1: 53.6 ± 11.0 C1: 50.3 ± 8.5
Liu et al. (37)	China	22	23	Randomized double-blind placebo trial	HD	I1: Probiotic capsule (2.2 ×10^9^cfu Balonium NQ1501, 0.53 ×10^9^cfu.L. acidophilus YIT2004, and 1.1 ×10^9^cfu E. faecalis YIT0072),8 capsule/d C1: Placebo capsules (pregelatinized starch and lactose), 8 capsule/d	24 w	23	22	I1:49 ± 9 C1:48 ± 11
Pan et al. (42)	China	50	48	Randomized controlled trial	PD	I1: Probiotic capsules (Bifidobacterium longum, Lactobacillus bulgaricus, and Streptococcus thermophilus), 6 capsules/d C1: Maltodextrin capsules, 6 capsules/d	8 w	56	42	I1: 49.31 ± 13.13 C1:50.92 ± 17.60
Natarajan et al. (21)	America	19	18	Randomized, double-blind, placebo-controlled crossover trial	HD	I1: Probiotic capsule (30 billion CFU of S. thermophilus KB 19, L. acidophilus KB 27, and B. longum KB 31), 6 capsules/d C1: Placebo capsules (a 1:1 blend of cream of wheat and psyllium husk)/d	24 w	6	16	I1:54 ± 39.62 C1:NA
Eidi et al. (29)	Iran	21	21	Randomized triple-blind placebo-controlled trial	HD	I1: Probiotic capsule (1.6 ×10^7^ CFU of Lactobacillus Rhamnoses), one capsule/d C1: Placebo capsule, one capsule/d	4 w	32	10	I1: 57.05 ± 13.95 C159.67 ± 15.04
Soleimani et al. (15)	Iran	30	30	Randomized, double-blind, placebo-controlled clinical trial	HD	I1: Synbiotic capsule (Lactobacillus acidophilus, Lactobacillus casei, and Bifidobacterium bifidum (2 ×10^9^ CFU/day each) plus 0.8 g/day of inulin) CI: Placebo (corn starch)	12 w	42	18	I1: 62.8 ± 12.7 C1: 62.8 ± 14.8
Viramontes-Horner et al. (23)	Mexico	20	15	Randomized double-blind, placebo-controlled, clinical trial	HD	I1: Symbiotic gel (Nutrihealth,Nutriments Inteligentes, S.A. de C.V, Guadalajara, Jalisco, Mexico) contained a mix of probiotics and 2.31 g of a prebiotic fiber(inulin); 1.5 g of omega-3 fatty acids and vitamins),14 gels/d CI: Placebo,14 gels/d	8 w	32	10	I1: 40.6 ± 17.1 C1: 39.0 ± 16.0
Lopes et al. (35)	Brazil	29	29	Randomized, simple-blind, placebo-controlled trial	HD	I1: Synbiotic drink (100 ml probiotic and 40 g of extruded sorghum flakes) C1: Placebo drink (100 mL of pasteurized milk and 40 g of extruded corn flakes)	7 w	38	20	I1:63.17 ± 11.16 C1:63.03 ± 10.77
Haghighat et al. (31)	Iran	I1:23I2:23	19	Randomized, double-blind, parallel group, placebo-controlled trial	HD	I1: Synbiotic sachet (5 g probiotics and 15 g of prebiotics), 20 g/d I2: Probiotic powder (5 g probiotics and 15 g of maltodextrin powder), 20 g/d C1: Maltodextrin powder, 20 g/d	12 w	34	31	I1: 48.04 ± 10.11 I2: 46.21 ± 11.49 C1:45.47 ± 10.76
Kooshki et al. (33)	Iran	23	23	Randomized, double-blind, placebo-controlled trial	HD	I1: Synbiotic capsules (100 mg of lactol probiotic, which contains Lactobacillus coagulant and fructo-oligosaccharides), 2 capsules/d C1: Placebo capsules (farina), 2 capsules/d	8 w	21	25	I1: 62.92 ± 16.80 C1:62.83 ± 16.62
Cruz-Mora et al. (20)	Mexico	8	10	Randomized, double-blind, placebo-controlled clinical trial	HD	I1: Symbiotic gel (probiotic of 2.0 3 ×10^12^ colony-forming units; 2.31 g of a prebiotic fiber (inulin); 1.5 g of omega-3 fatty acids (eicosatetraenoic and docosahexaenoic acid) and vitamins (complex B, folic acid, ascorbic acid, and vitamin E) C1: Placebo gel (a gel without prebiotic fiber, probiotics, omega-3 fatty acids, vitamins)	8 w	15	3	I1:34 ± 10 C1:30.6 ± 9.5
Mirzaeian et al. (38)	Iran	21	21	Randomized, double-blind, placebo-controlled clinical trial	HD	I1: Synbiotic capsule (Lactobacillus casei L.acidophilus Rhamnoses, Bulgaricus, Bifidobacterium breve, B. longum and Streptococcus thermophiles and fructo-oligosaccharide as prebiotic in addition to lactose, magnesium stearate, and talc as filling materials), 1 g/d CI: Placebo capsules (maltodextrin), 1 g/d	8 w	30	12	I1:58.30 ± 11.3 C1:69.74 ± 42.87

*I, intervention; C, control; RCT, randomized clinical trial; HD, hemodialysis; PD, peritoneal dialysis; M, male; F, female; W, week; NA, not available*.

In the published article, there was an error in Supplementary Figure 6. Panel A (BUN) is the same as panel B (Creatinine). The correct material statement appears below.

Supplementary Figure 6 Results of direct comparisons for other clinical outcomes. Forest plot of the effect of prebiotic, probiotic, and synbiotic supplementation on (A) BUN (mg/dl); (B) Creatinine (mg/dl); (C) Urea (mg/dl); (D) Uric acid (mg/dl).

In the published article, there was an error in Supplementary Figure 15. The figure legend of (E) p-cresyl sulfate (PCS) should be replaced with (E) Malondialdehyde (MDA), and the original material statement was Supplementary Figure 15. Sensitivity analysis. Sensitivity analysis for the network of (A) C-reactive protein (CRP); (B) Interleukin-6(IL-6); (C) tumor necrosis factor-α (TNF-α); (D) Indoxyl sulfate (IS); (E) p-cresyl sulfate (PCS); (F) Urea. The correct material statement appears below.

Supplementary Figure 15 Sensitivity analysis. Sensitivity analysis for the network of (A) C-reactive protein (CRP); (B) Interleukin- 6(IL-6); (C) tumor necrosis factor-α (TNF-α); (D) Indoxyl sulfate (IS); (E) Malondialdehyde (MDA); (F) Urea.

In the published article, there was an error. “IAA” should be changed to “MDA,” “96.8%” should be changed to “95%,” “Synbiotics” should be changed to “Probiotics,” “MDA” should be changed to “IAA,” “95.6%” should be changed to “86.3%” in the Results. A correction has been made to **Results**, *Network Meta-Analysis*, Uremic Toxins. The corrected sentence appears below:

“Uremic toxins including IS, PCS, IAA, and MDA were evaluated. The outcome revealed prebiotics were superior in declining IS (prebiotics: SMD −0.43; 95% CI [−0.81, −0.05]), prebiotics and synbiotics were effective supplements on the alteration of MDA level (prebiotics: SMD −1.88; 95% CI [−3.02, −0.75]; synbiotics: SMD −0.85; 95% CI [−1.67, −0.02]) but no supplements significantly declined serum PCS, and IAA (Figure 5 and Supplementary Figure 11). With regard to IS, PCS, and MDA, prebiotics were ranked as the first therapeutic option, where the SUCRA were 84.7, 77, and 95%, respectively. Probiotics had the highest possibility in serum IAA level (SUCRA = 86.3%) (**Figure 6**).”

In the published article, there was an error. “Synbiotics” should be changed to “prebiotics” in the Discussion section, and “respectively” needed to be removed from another sentence. A correction has been made to **Discussion**, Paragraph 3. The corrected sentence appears below:

“Prebiotics were superior in reducing serum IS, prebiotics were rated as best in reducing MDA level. The accumulation of metabolic toxins in the blood is closely associated with the deteriorating progression of CKD to ESRD, part of the toxins, such as protein-bound uremic toxins, come from intestinal flora, and dialysis is not potentially removed (9, 45). The efficacy of pro/pre/syn-biotics in lowering uremic toxins has been demonstrated by previous meta-analysis (13, 49). Our pairwise comparison found the same results and notably we further suggested that prebiotics and synbiotics are the most effective supplements. Prebiotics are some non-digestible food ingredients, regarded as a vital dietary supplement for ESRD patients with dietary restriction of protein intake, increasing the concentration of short-chain fatty acids (SCFAs), which benefit metabolites produced by gut bacterium (12, 50). Decreased SCFAs were regarded as one of the main mechanisms of the production of uremic toxins, which may also be the reason why prebiotics were more effective than probiotics and synbiotics. MDA is a low-molecular-weight solution that participates in oxidative stress, connecting with the progress of CKD and its cardiovascular complications (51). Seven randomized controlled trials were introduced in the study of Nguyen et al. (14), who found that MDA was significantly reduced in hemodialysis patients after taking three supplements. Several studies also have demonstrated that synbiotics might increase the expression of the antioxidant gene SOD and GPX in the gut by targeting gut bacteria to activate oxidative stability (52, 53). Current studies support the evidence that taking prebiotics and synbiotics have the most beneficial influence in reducing IS and MDA. Whereas, it is of great importance to emphasize that the change of uremic toxins is the result of multiple comparisons among the three drugs, combining small samples of studies and different follow-up times, which declined the strength of evidence, contributing to the accuracy of evidence is low. Thus, launching large clinical trials is important to evaluate the function of pro/pre/syn-biotics in reducing uremic toxins, especially protein-bound uremic toxins.”

The authors apologize for these errors and state that this does not change the scientific conclusions of the article in any way. The original article has been updated.

## Publisher's note

All claims expressed in this article are solely those of the authors and do not necessarily represent those of their affiliated organizations, or those of the publisher, the editors and the reviewers. Any product that may be evaluated in this article, or claim that may be made by its manufacturer, is not guaranteed or endorsed by the publisher.

