# Supplementary material for: Probiotics, Prebiotics, and Synbiotics Improve Uremic, Inflammatory, and Gastrointestinal Symptoms in End-Stage Renal Disease With Dialysis: A Network Meta-Analysis of Randomized Controlled Trials
Source: Front Nutr. 2022 Apr 4;9:850425. doi: 10.3389/fnut.2022.850425 (PMC9015659; doi:10.3389/fnut.2022.850425)
Supplement: Supplementary file 1 [file Data_Sheet_1.pdf]

Supplement Material

1 Supplementary Figures

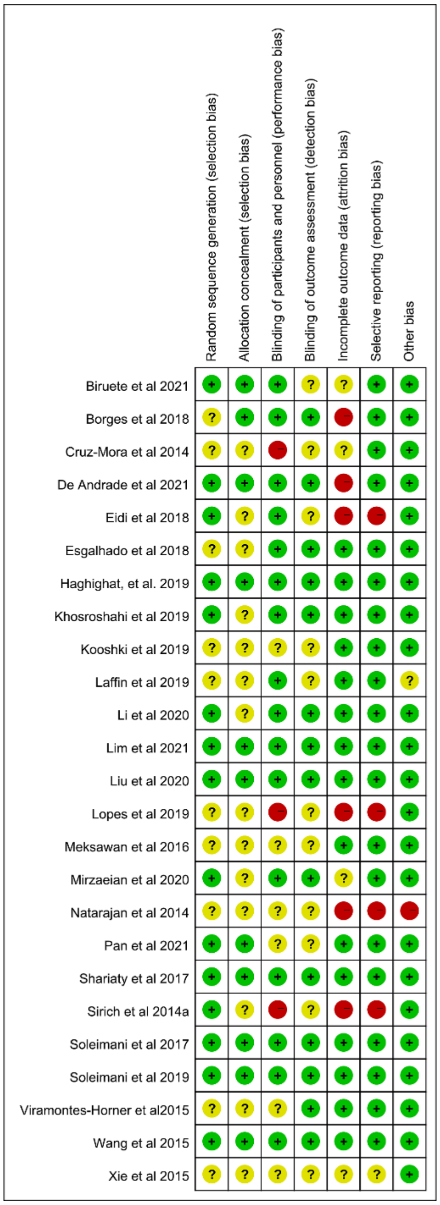

Supplementary Figure 1. Risk of bias summary across the included studies. Unclear risk of bias: ‘‘?’’, low risk of bias: ‘‘+’’, and high risk of bias: ‘‘-’’.

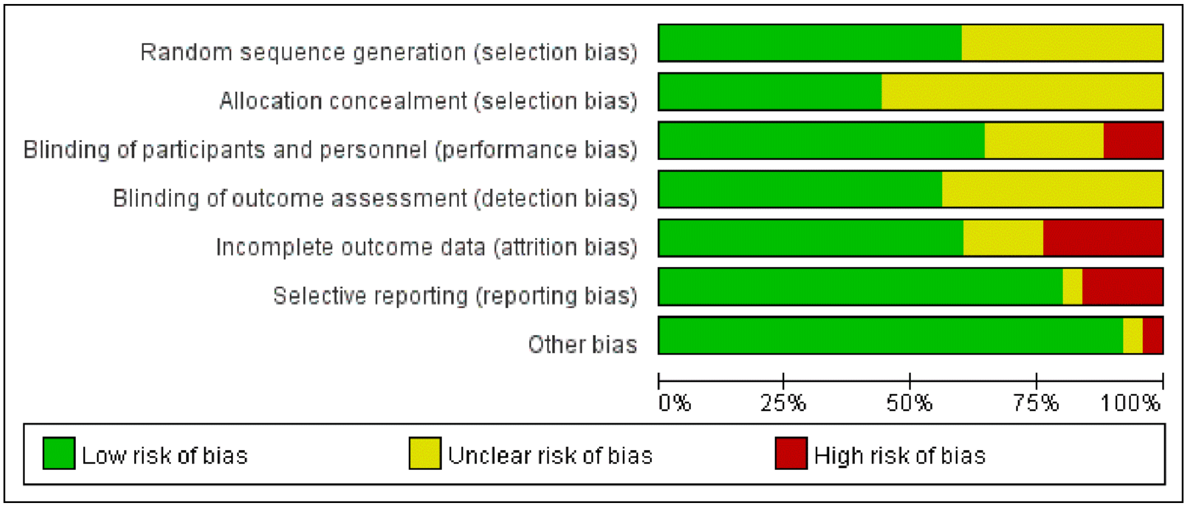

Supplementary Figure2. Graph of Risk of bias**.** Risk of bias graph across the included studies. Unclear risk of bias: ‘‘yellow’’, low risk of bias: ‘‘green’’, and high risk of bias: ‘‘red’’.

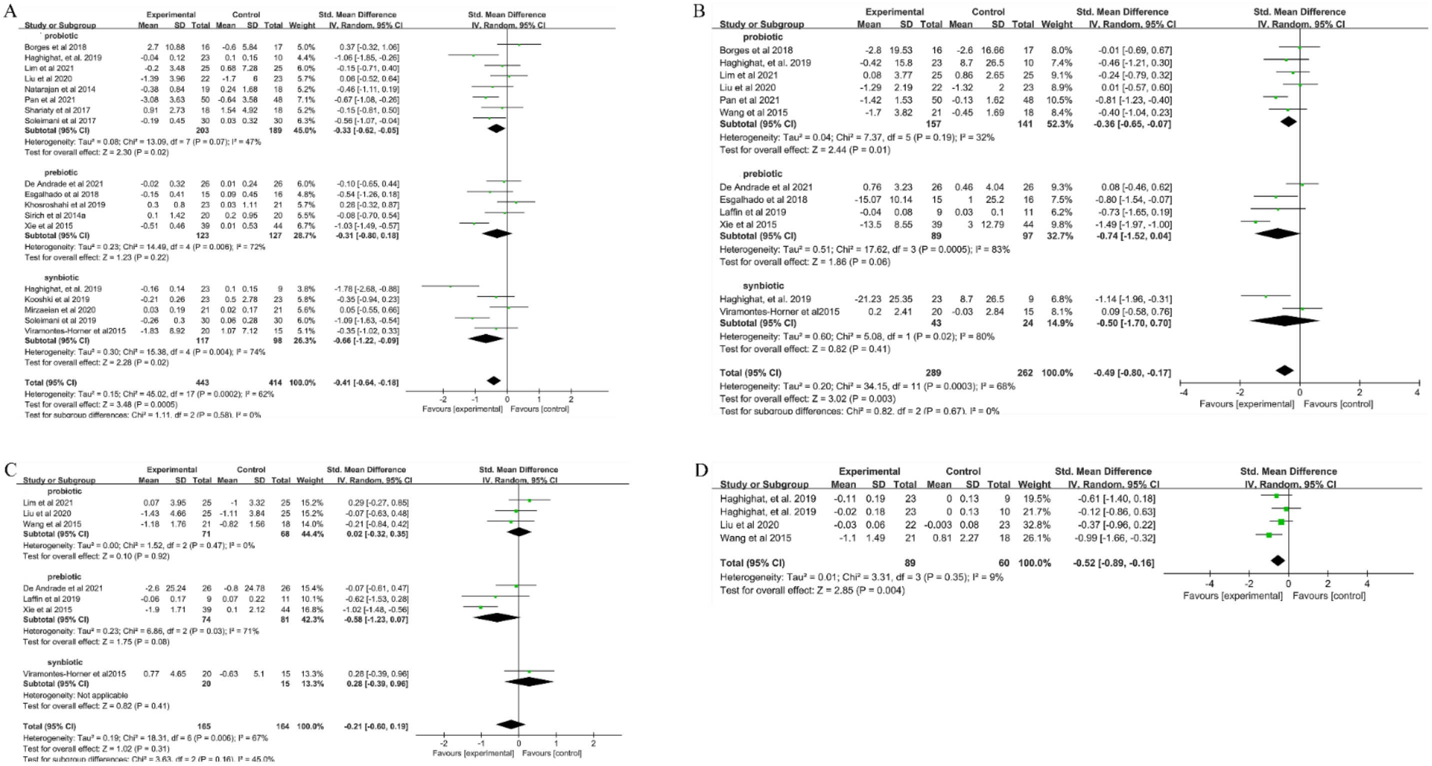

Supplementary Figure3. Results of direct comparisons for inflammatory factors**.** Forest plot of the effect of prebiotic, probiotic, and synbiotic supplements on (A) C-reactive protein (CRP, mg/dl); (B)Interleukin- 6(IL-6, pg/ml); (c) tumor necrosis factor-α (TNF-α, pg/ml); (D) endotoxin (IU/L)

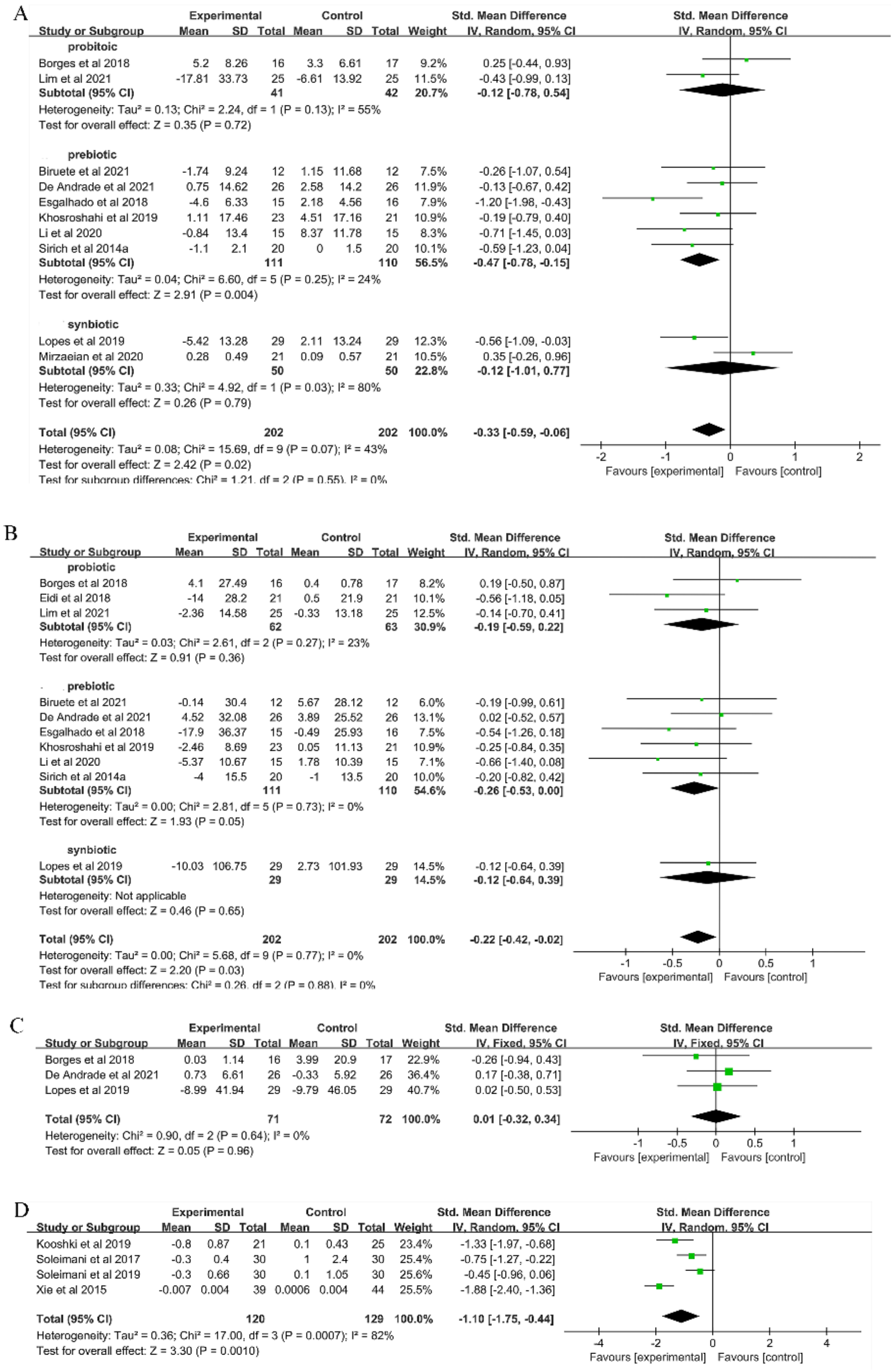

Supplementary Figure4. Results of direct comparisons for uremic toxins. Forest plot of the effect of prebiotic, probiotic, and synbiotic supplementation on (A)serum Indoxyl sulfate (IS, mg/L); (B)p-cresyl sulfate (PCS, mg/L); (C) Indole-3-acetic acid (IAA, umol/L); (D) Malondialdehyde (MDA, umol/L).

Study or Subgroup	Experimental Mean	Experimental SD	Experimental Total	Control Mean	Control SD	Control Total	Weight	Std. Mean Difference IV, Fixed, 95% CI
Cruz-Mora et al 2014	-3	3.09	8	-1.66	9.69	10	15.7%	-0.17 [-1.10, 0.76]
Haghighat, et al. 2019	-0.65	3.24	23	0.42	3.31	9	22.7%	-0.32 [-1.10, 0.46]
Haghighat, et al. 2019	-0.79	2.73	23	0.42	3.31	10	24.2%	-0.41 [-1.16, 0.34]
Liu et al 2020	-2.9	2.5	22	-0.83	3.37	23	37.5%	-0.68 [-1.29, -0.08]
**Total (95% CI)**			**76**			**52**	**100.0%**	**-0.45 [-0.82, -0.08]**

Heterogeneity: Chi² = 1.04, df = 3 (P = 0.79); I² = 0%
Test for overall effect: Z = 2.41 (P = 0.02)

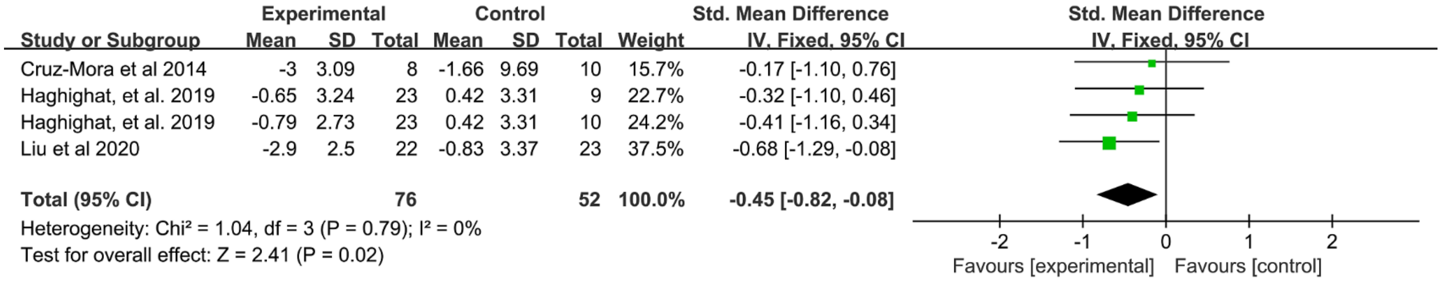

Supplementary Figure5. Results of direct comparisons for GI symptoms. Forest plot of the effect of prebiotic, probiotic, and synbiotic supplementation on gastrointestinal-symptoms (GI symptoms)

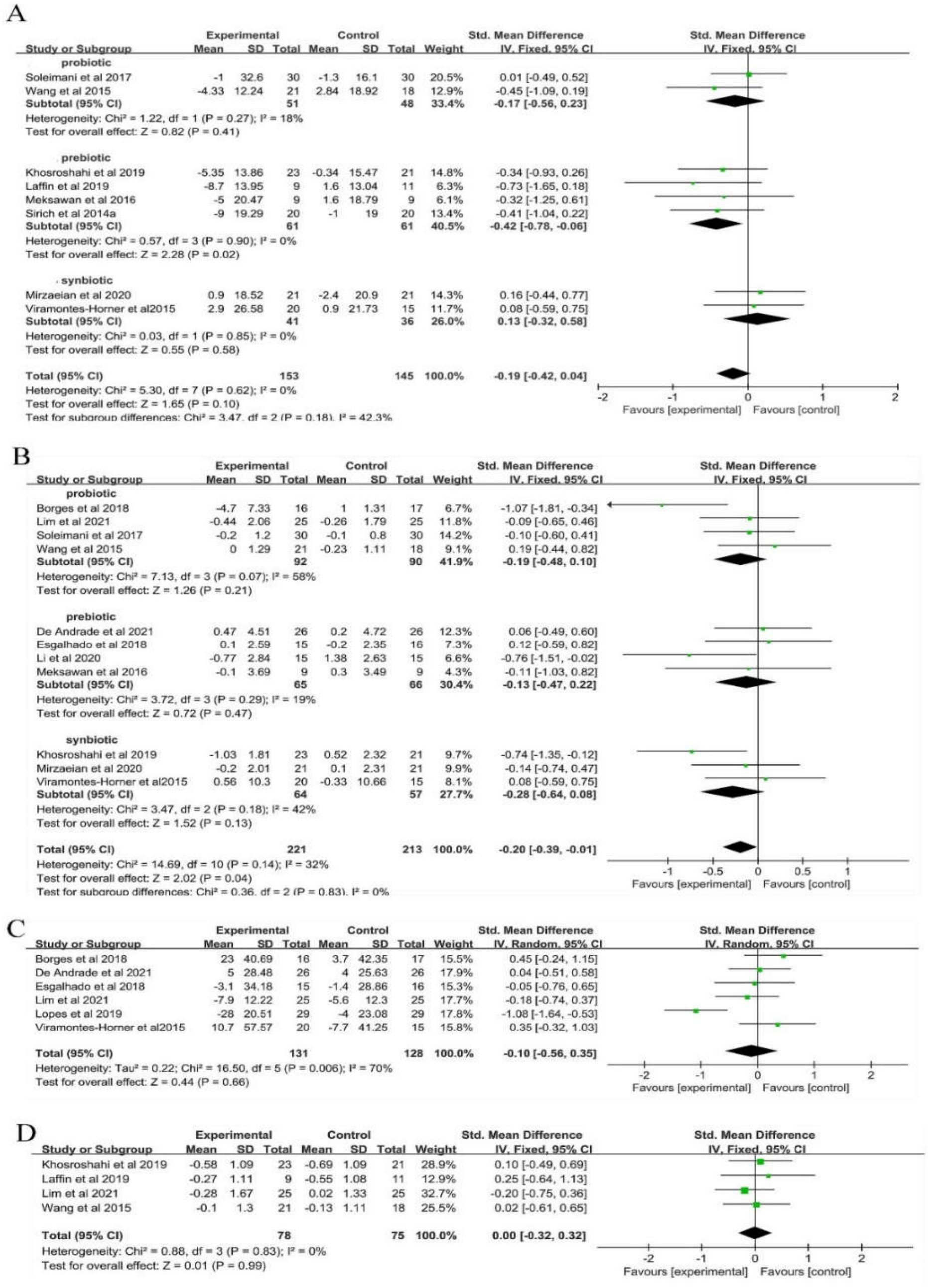

Supplementary Figure6. Results of direct comparisons for other clinical outcomes. Forest plot of the effect of prebiotic, probiotic, and synbiotic supplementation on (A) BUN (mg/dl); (B) Creatinine (mg/dl); (C) Urea (mg/dl); (D) Uric acid (mg/dl).

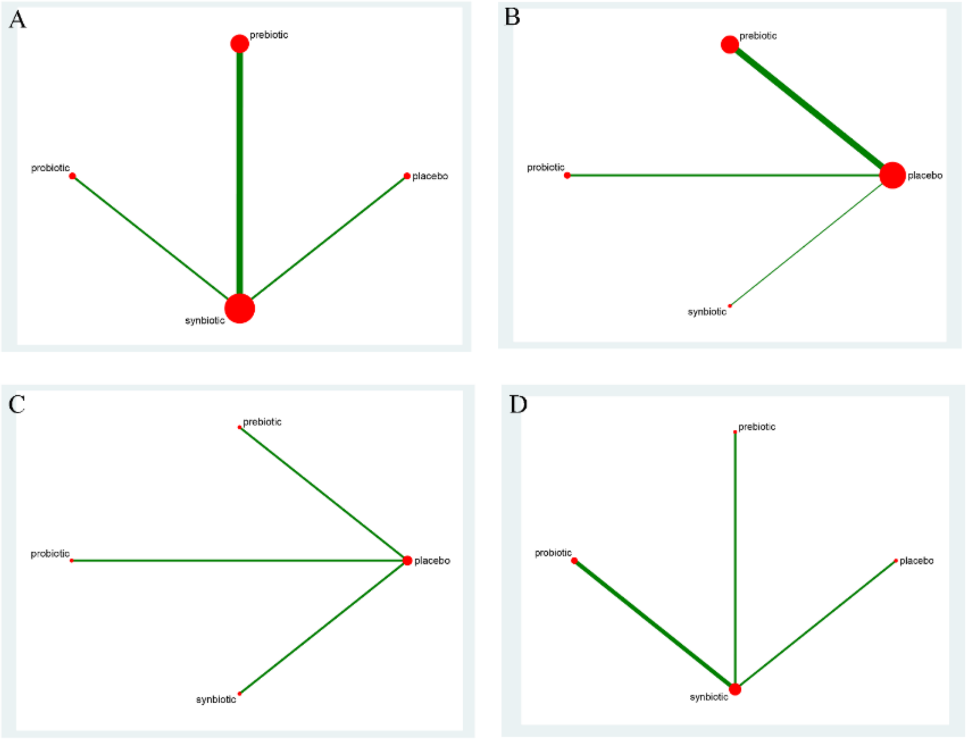

Supplementary Figure7. Network graph of all treatments in uremic toxins. Outcome: Indoxyl sulfate (IS); (B)p-cresyl sulfate (PCS); (C) Indole-3-acetic acid (IAA); (D) Malondialdehyde (MDA). The number of studies for each treatment can be indicated by the size of each circle. Direct comparisons of tests can be expressed by lines between nodes, and the number of tests connected to the network can be expressed by the thickness of the lines.

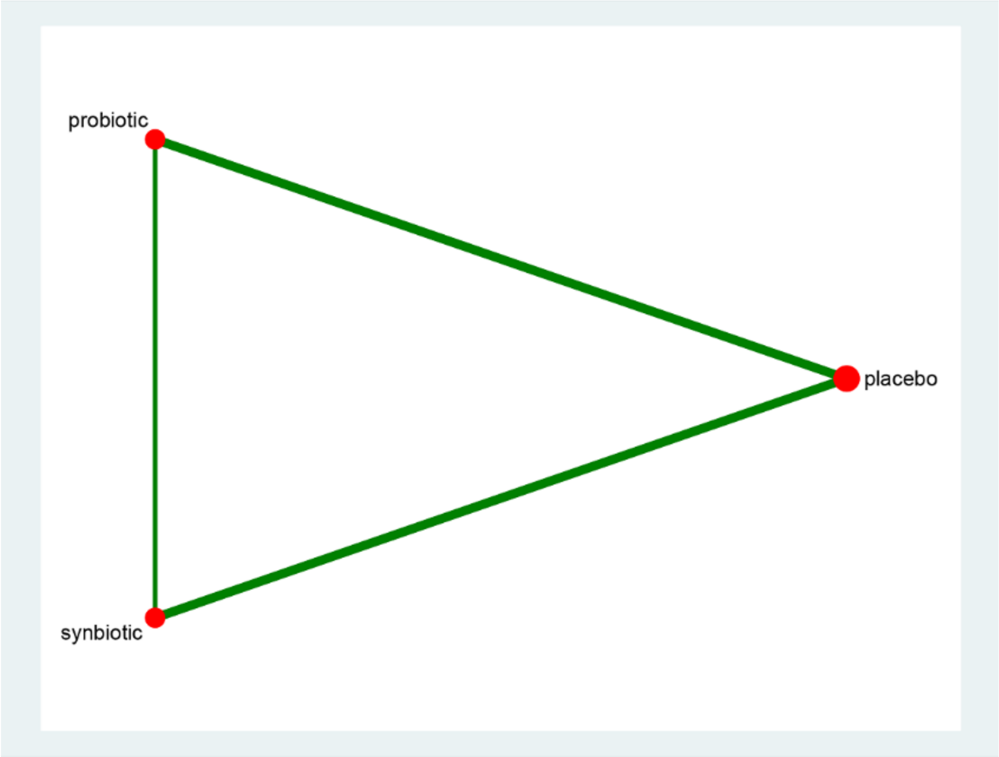

Supplementary Figure8. Network graph of all treatments in gastrointestinal symptoms. Outcome: gastrointestinal-symptoms (GI symptoms). The number of studies for each treatment can be indicated by the size of each circle. Direct comparisons of tests can be expressed by lines between nodes, and the number of tests connected to the network can be expressed by the thickness of the lines.

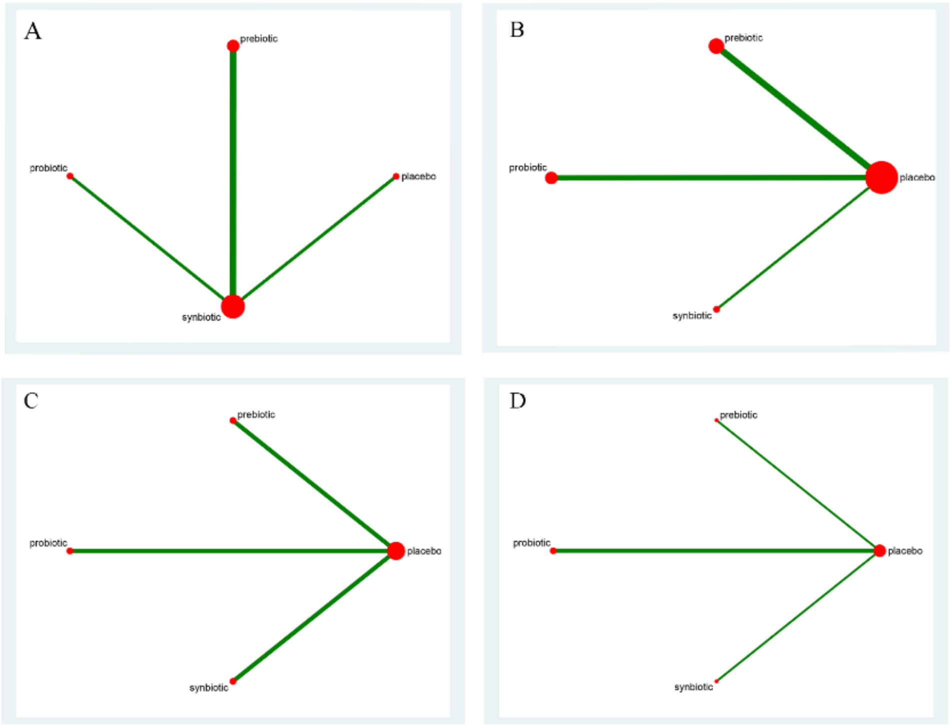

Supplementary Figure9. Network graph of all treatments in other clinical outcomes.Outcome:(A) BUN;(B) Creatinine;(C) Urea;(D) Uric acid. The number of studies for each treatment can be indicated by the size of each circle. Direct comparisons of tests can be expressed by lines between nodes, and the number of tests connected to the network can be expressed by the thickness of the lines.

A

placebo	**-0.74 (-1.32, -0.16) ***	-0.26 (-0.71,0.19)	-0.68 (-1.44,0.09)
0.31 (-0.14,0.76)	prebiotic	0.48 (-0.25,1.21)	0.07 (-0.89,1.03)
0.27 (-0.08,0.62)	-0.04 (-0.62,0.54)	probiotic	-0.41 (-1.23,0.40)
**0.69 (0.24,1.14) ***	0.38 (-0.26,1.02)	0.42 (-0.12,0.96)	synbiotic

B

placebo			
**0.59 (0.08,1.09) ***	prebiotic		
0.01 (-0.49,0.51)	-0.58 (-1.29,0.13)	probiotic	
-0.28 (-1.21,0.64)	-0.87 (-1.92,0.18)	-0.29 (-1.34,0.76)	synbiotic

C

placebo		
**0.46 (0.10,0.82) ***	probiotic	
**0.83 (0.27,1.38) ***	0.37 (-0.18,0.91)	synbiotic

Supplementary Figure10. Network meta-analysis of all treatments on uremic toxins.A pair comparison from network meta-analysis on root coverage procedures, for Changes in: (A) C-reactive protein (CRP, lower) and Interleukin- 6 (IL-6, upper); (B) tumor necrosis factor-α (TNF-α, lower); (C) endotoxin(lower). The column-defining intervention was compared with the row-defining intervention. The result is an estimate standard Mean difference (SMD) and 95% confidence interval (CI) from the NMA model is in a common cell.

A

placebo	-0.26 (-0.53,0.00)	-0.19 (-0.55,0.16)	-0.12 (-0.64,0.39)
**0.43 (0.05,0.81) ***	prebiotic	0.07 (-0.37,0.51)	0.14 (-0.44,0.72)
0.12 (-0.50,0.75)	-0.31 (-1.04,0.42)	probiotic	0.07 (-0.55,0.70)
0.14 (-0.46,0.73)	-0.29 (-1.00,0.41)	0.01 (-0.85,0.87)	synbiotic

B

placebo	**-1.88 (-3.02, -0.75) ***	-0.75 (-1.88,0.39)	**-0.85 (-1.67, -0.02) ***
-0.17 (-0.71,0.38)	prebiotic	1.14 (-0.47,2.74)	1.04 (-0.37,2.44)
0.38 (-0.31,1.07)	0.54 (-0.33,1.42)	probiotic	-0.10 (-1.50,1.30)
-0.02 (-0.53,0.50)	0.15 (-0.60,0.90)	-0.40 (-1.26,0.46)	synbiotic

Supplementary Figure11. Network meta-analysis of all treatments on uremic toxins. A pair comparison from network meta-analysis on root coverage procedures, for Changes in: (A) Indoxyl sulfate (IS,lower) and p-cresyl sulfate (PCS, upper); (B) Indole-3-acetic acid (IAA, lower) and Malondialdehyde (MDA, upper). The column-defining intervention was compared with the row-defining intervention. The result is an estimate standard Mean difference (SMD) and 95% confidence interval (CI) from the NMA model is in a common cell.

placebo		
**0.52 (0.10,0.93) ***	probiotic	
0.36 (-0.12,0.84)	-0.16 (-0.67,0.35)	synbiotic

Supplementary Figure12. Network meta-analysis of all treatments on GI symptoms. A pair comparison from network meta-analysis on root coverage procedures, for Changes in GI symptoms (lower). The column-defining intervention was compared with the row-defining intervention. The result is an estimate standard Mean difference (SMD) and 95% confidence interval (CI) from the NMA model is in a common cell.

A

placebo	-0.28 (-0.68,0.11)	-0.22 (-0.63,0.19)	-0.03 (-0.63,0.56)
**0.42 (0.06,0.78) ***	prebiotic	0.06 (-0.50,0.63)	0.25 (-0.46,0.96)
0.17 (-0.23,0.56)	-0.25 (-0.79,0.28)	probiotic	0.19 (-0.53,0.91)
-0.13 (-0.58,0.32)	-0.55 (-1.12,0.03)	-0.29 (-0.89,0.31)	synbiotic

B

placebo	0.25 (-0.64,1.13)	-0.10 (-0.52,0.32)	0.10 (-0.49,0.69)
0.01 (-0.89,0.90)	prebiotic	-0.34 (-1.32,0.63)	-0.15 (-1.21,0.92)
-0.12 (-1.01,0.78)	-0.12 (-1.39,1.14)	probiotic	0.20 (-0.53,0.92)
0.40 (-0.49,1.29)	0.40 (-0.87,1.66)	0.52 (-0.74,1.78)	synbiotic

Supplementary Figure13. Network meta-analysis of all treatments on other clinical outcomes. A pair comparison from network meta-analysis on root coverage procedures, for Changes in (A): BUN (lower) and Creatinine (upper); (B): Urea (lower) and Uric acid (upper). The column-defining intervention was compared with the row-defining intervention. The result is an estimate standard Mean difference (SMD) and 95% confidence interval (CI) from the NMA model is in a common cell.

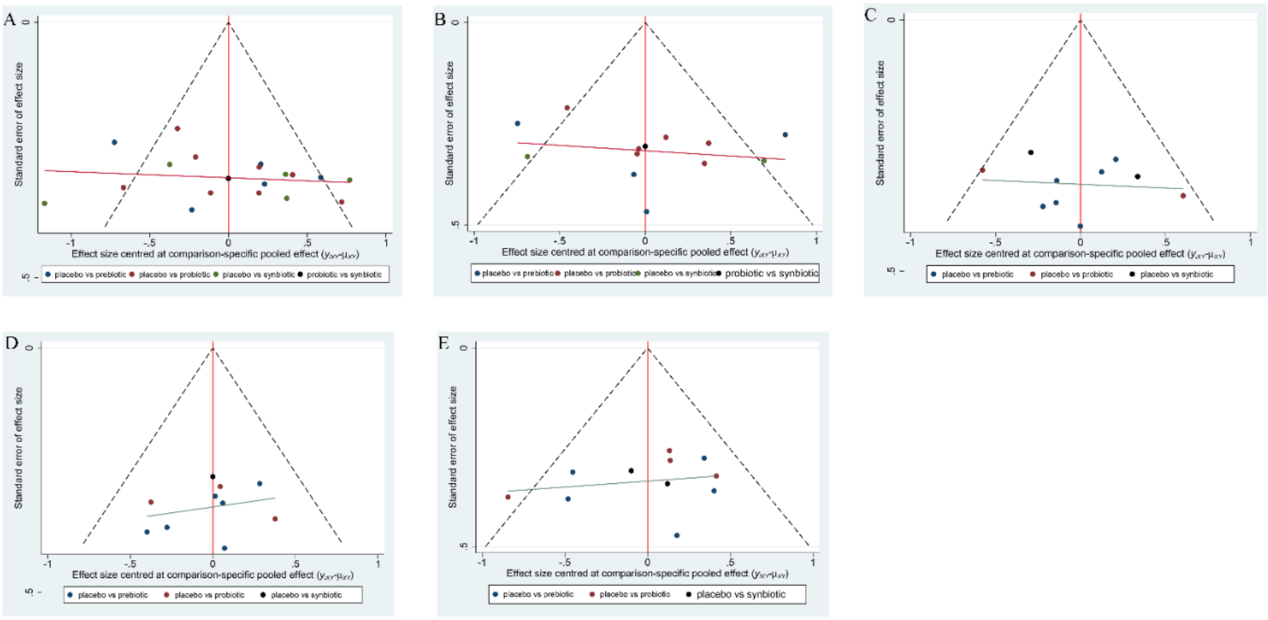

Supplementary Figure14. Funnel plot. Funnel plot for the network of (A) C-reactive protein (CRP); (B) Interleukin- 6(IL-6); (C) Indoxyl sulfate (IS); (D) p-cresyl sulfate (PCS); (E) Creatinine.

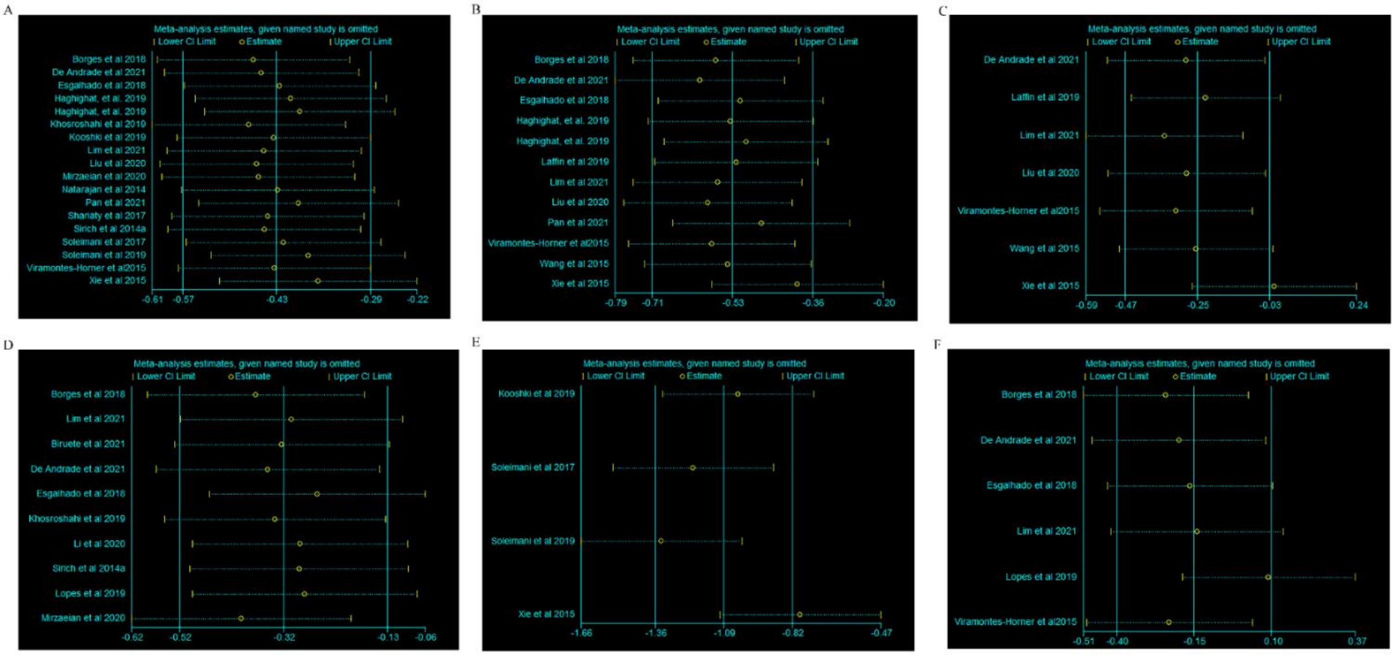

Supplementary Figure 15. Sensitivity analysis. Sensitivity analysis for the network of (A) C-reactive protein (CRP); (B) Interleukin- 6 (IL-6); (C) tumor necrosis factor-α (TNF-α); (D) Indoxyl sulfate (IS); (E) Malondialdehyde (MDA); (F) Urea.
