# Supplementary material for: Probiotics, Prebiotics, and Synbiotics Improve Uremic, Inflammatory, and Gastrointestinal Symptoms in End-Stage Renal Disease With Dialysis: A Network Meta-Analysis of Randomized Controlled Trials
Source: Front Nutr. 2022 Apr 4;9:850425. doi: 10.3389/fnut.2022.850425 (PMC9015659; doi:10.3389/fnut.2022.850425)
Supplement: Supplementary file 2 [file Table_1.DOCX]

Supplement Material

# Supplementary Tables

### Supplement Table 1. Search strategy

| Data base | Search Term |
| --- | --- |
| Pubmed | #1: ("Probiotics"[MeSH Terms] OR "Lactobacillus"[MeSH Terms] OR "Bifidobacterium"[MeSH Terms] OR ("probiotic*"[Title/Abstract] OR "Lactobacillus"[Title/Abstract] OR "Bifidobacterium"[Title/Abstract]) OR "Synbiotics"[MeSH Terms] OR "synbiotic*"[Title/Abstract] OR "Prebiotics"[MeSH Terms] OR "prebiotic*"[Title/Abstract])  #2: ("Kidney Diseases"[MeSH Terms] OR "Renal Replacement Therapy"[MeSH Terms] OR "kidney failure, chronic"[MeSH Terms] OR "Renal Dialysis"[MeSH Terms] OR "Uremia"[MeSH Terms] OR "Hemofiltration"[MeSH Terms] OR "Peritoneal Dialysis"[MeSH Terms] OR ("end stage renal disease"[Title/Abstract] OR "end stage kidney disease"[Title/Abstract] OR "endstage renal disease"[Title/Abstract] OR "endstage kidney disease"[Title/Abstract] OR "ESRD"[Title/Abstract] OR "ESRF"[Title/Abstract] OR "ESKD"[Title/Abstract] OR "ESKF"[Title/Abstract]) OR ("Hemodialysis"[Title/Abstract] OR "haemodialysis"[Title/Abstract] OR "hemodiafiltration"[Title/Abstract] OR "haemodiafiltration"[Title/Abstract] OR "kidney artificial"[Title/Abstract] OR "predialysis"[Title/Abstract] OR "ultrafiltration"[Title/Abstract]) OR ("ultrafilt*"[Title/Abstract] OR "dialy*"[Title/Abstract] OR "biofilt*"[Title/Abstract] OR "uremi*"[Title/Abstract] OR "uraemia*"[Title/Abstract]))  #3: (("randomized controlled trial"[Publication Type] OR "controlled clinical trial"[Publication Type] OR "randomized"[Title/Abstract] OR "placebo"[Title/Abstract] OR "clinical trials as topic"[MeSH Terms] OR "randomly"[Title/Abstract] OR "trial"[Title]) NOT ("animals"[MeSH Terms] NOT "humans"[MeSH Terms]))  #4: #1AND #2 AND #3 |
| Embase | #1: 'probiotic agent'/exp OR 'lactobacillus'/exp OR 'bifidobacterium'/exp OR 'synbiotic agent'/exp OR 'prebiotic agent'/exp OR probiotic*:ab,ti OR lactobacillus:ab,ti OR bifidobacterium:ab,ti OR synbiotic*:ab,ti OR prebiotic*:ab,ti  #2: 'kidney disease'/exp OR 'renal replacement therapy'/exp OR 'chronic kidney failure'/exp OR 'hemodialysis'/exp OR 'peritoneal dialysis'/exp OR 'uremia'/exp OR 'hemofiltration'/exp OR 'end stage renal disease'/exp OR 'end stage renal disease':ab,ti OR 'end stage kidney disease':ab,ti OR 'endstage renal disease':ab,ti OR 'endstage kidney disease':ab,ti OR eskd:ab,ti OR esrd:ab,ti OR esrf:ab,ti OR eskf:ab,ti OR predialysis:ab,ti OR 'kidney artificial':ab,ti OR 'ultrafiltration':ab,ti OR ultrafilt*:ab,ti OR dialy*:ab,ti OR hemodialysis:ab,ti OR haemodialysis:ab,ti OR hemodiafiltration:ab,ti OR haemodiafiltration:ab,ti OR biofilt*:ab,ti OR uremi*:ab,ti OR uraemia*:ab,ti  #3: ('crossover procedure':de OR 'double-blind procedure':de OR 'randomized controlled trial':de) AND or  AND 'single-blind procedure':de OR (random*:de,ab,ti AND or :de,ab,ti AND factorial*:de,ab,ti) OR crossover*:de,ab,ti OR ((cross NEXT/1 over*):de,ab,ti) OR placebo*:de,ab,ti OR ((doubl*NEAR/1 blind*):de,ab,ti) OR ((singl* NEAR/1 blind*):de,ab,ti) OR assign*:de,ab,ti OR allocat*:de,ab,ti OR volunteer*:de,ab,ti  #4: #1 AND #2 AND #3 |
| The Cochrane Register of Controlled Trials | #1: MeSH descriptor: [Probiotics] explode all trees OR MeSH descriptor: [Lactobacillus] explode all trees OR MeSH descriptor: [Bifidobacterium] explode all trees OR MeSH descriptor: [Synbiotics] explode all trees OR MeSH descriptor: [Prebiotics] explode all trees OR (probiotic*):ti,ab,kw OR (Lactobacillus):ti,ab,kw OR (Bifidobacterium):ti,ab,kw OR (synbiotic*):ti,ab,kw OR (prebiotic*):ti,ab,kw (Word variations have been searched)  #2: MeSH descriptor: [Kidney Diseases] explode all trees OR MeSH descriptor: [Renal Replacement Therapy] explode all trees OR MeSH descriptor: [Kidney Failure, Chronic] explode all trees OR MeSH descriptor: [Renal Dialysis] explode all trees OR MeSH descriptor: [Uremia] explode all trees OR MeSH descriptor: [Hemofiltration] explode all trees OR MeSH descriptor: [Peritoneal Dialysis] explode all trees OR ("end stage renal disease"):ti,ab,kw OR ("end stage kidney disease"):ti,ab,kw OR ("endstage renal disease"):ti,ab,kw OR ("endstage kidney disease"):ti,ab,kw (Word variations have been searched) OR (ESRD):ti,ab,kw OR (ESRF):ti,ab,kw OR (ESKD):ti,ab,kw OR (ESKF):ti,ab,kw (Word variations have been searched) OR (Hemodialysis):ti,ab,kw OR (haemodialysis):ti,ab,kw OR (hemodiafiltration):ti,ab,kw OR (haemodiafiltration):ti,ab,kw (Word variations have been searched) OR (predialysis):ti,ab,kw OR (ultrafiltration):ti,ab,kw OR (kidney artificial):ti,ab,kw OR (ultrafilt*):ti,ab,kw OR (dialy*):ti,ab,kw (Word variations have been searched) OR (biofilt*):ti,ab,kw OR (uremi*):ti,ab,kw OR (uraemia*):ti,ab,kw (Word variations have been searched)  #3: #1 AND #2 |

### Supplement Table 2. Egger’s test

| **intervention** | **P-value** |
| --- | --- |
| C-reactive protein | 0.43 |
| Interleukin-6 | 0.42 |
| tumor necrosis factor-α | 0.49 |
| endotoxin | 0.15 |
| indoxyl sulfate | 0.52 |
| p-Cresyl sulfate | 0.25 |
| indole-3-acetic acid | 0.33 |
| malondialdehyde | 0.77 |
| blood urea nitrogen | 0.18 |
| creatinine | 0.35 |
| urea | 0.23 |
| uric acid | 0.25 |
| gastrointestinal symptoms | 0.41 |

Egger’s test of all outcomes, P>0.05 suggested no public bias.

### Supplement Table 3. Inconsistency evaluation

| **Index** | **Loop** | **IF** | **seIF** | **z_vaule** | **p_vaule** | **CI_95** | **Loop_Heterog_tau2** |
| --- | --- | --- | --- | --- | --- | --- | --- |
| CRP | placbo-probiotic-synbiotic | 0.406 | 0.64 | 0.635 | 0.525 | (0.00,1.66) | 0.189 |
| IL-6 | placbo-probiotic-synbiotic | 0.602 | 0.696 | 0.865 | 0.387 | (0.00,1.97) | 0.186 |
| endotoxin | placbo-probiotic-synbiotic | 0.527 | 0.49 | 1.077 | 0.281 | (0.00,1.49) | 0.000 |
| GI-symptoms | placbo-probiotic-synbiotic | 0.323 | 0.601 | 0.538 | 0.59 | (0.00,1.5) | 0.000 |
